# Recent advances in 1,3,4-thiadiazole-based anticancer agents: synthesis, molecular targets, docking studies, and structure–activity relationships

**DOI:** 10.1039/d6md00857g

**Published:** 2026-10-27

**Authors:** Glanish Jude Martis, Santosh L. Gaonkar

**Affiliations:** a Manipal Academy of Higher Education, Manipal Institute of Technology Manipal India sl.gaonkar@manipal.edu

## Abstract

1,3,4-Thiadiazole compounds have potential as anticancer agents. In recent years, they have been found to be promising inhibitors of some of the major classes of cancer mediators, such as tyrosine kinases and phosphatases. The inhibition of VEGFR-2 and EGFR kinases is pivotal for blocking the signaling pathways and inhibiting tumor growth. The SHP2 protein, a key mediator of tumor growth, tends to combat cell proliferation under different types of carcinomic conditions. Thiadiazole core molecules are instrumental in inhibiting such mediators and act as antiproliferative agents. This review focuses on the recent trends in thiadiazole chemistry and the synthesis of thiadiazole-containing heterocyclic compounds, along with their anticancer activity. An in-depth analysis of molecular docking studies and structure–activity relationships (SARs) provides strong evidence for the design of new thiadiazole-based drug molecules. Recent advances in the field of thiadiazole-containing heterocyclic systems in terms of their anticancer activity have been discussed with exclusive coverage over the past five years, since 2021.

## Abstract 2

1,3,4-Thiadiazole compounds have potential as anticancer agents.

## Graphical abstract

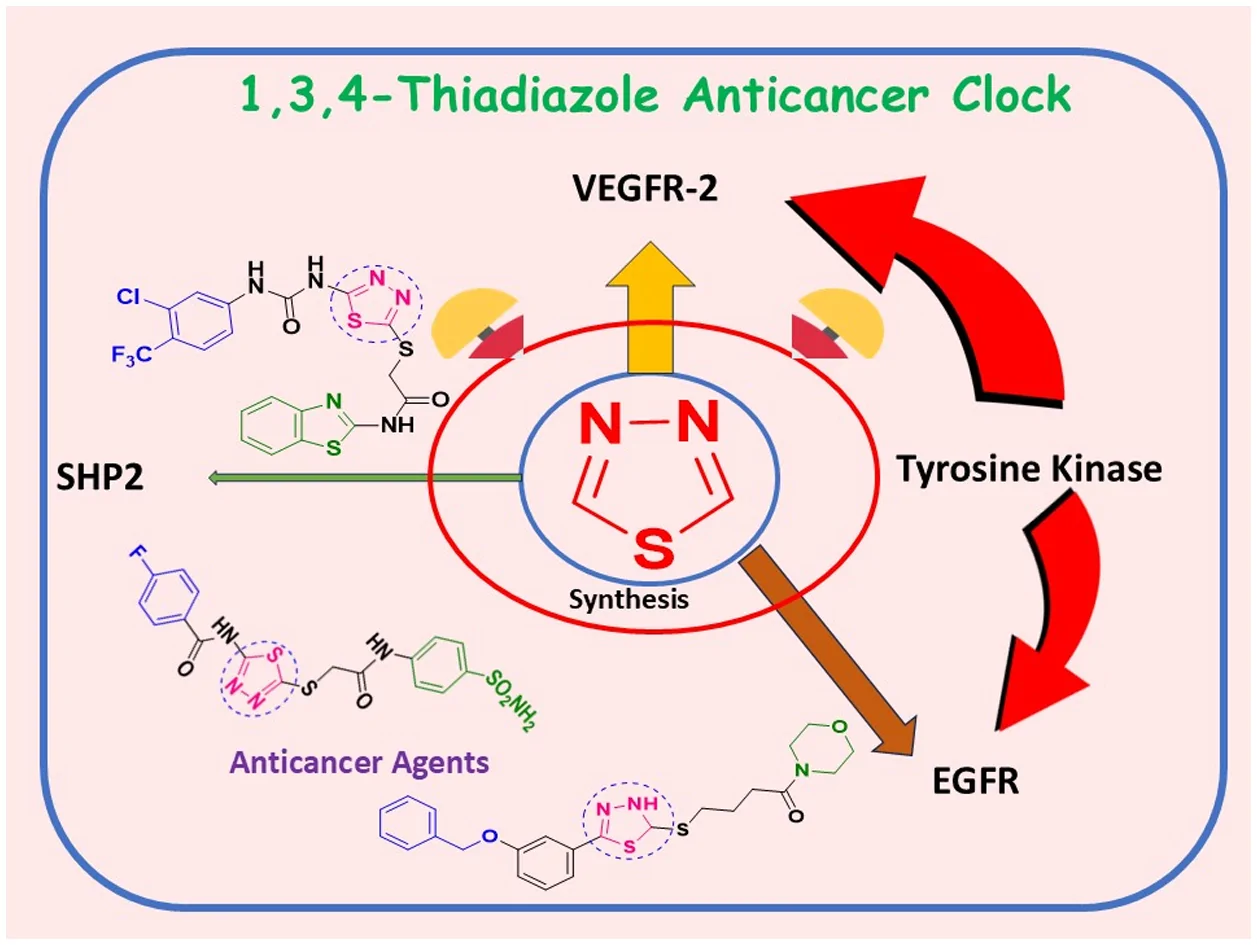

## 1. Introduction

Heterocyclic systems have created their own hallmark in indexing their potential and role in the field of medicinal chemistry, pharmaceutical innovation and drug discovery.^1–3^ Their synthesis and biological studies have been continuously advancing for three decades now.^4–6^ Especially, azoles have left an indelible mark in the fields of synthetic chemistry and drug development. For many years now, there has been a tremendous transition in producing azole-containing scaffolds.^7–9^ The impact of azole-containing heterocyclic hybrids in the market has reached a new trajectory in the economic strata.^10–14^ Thus, the scope for developing azole moieties is in great demand, and with this intent, 1,3,4-thiadiazoles have been a subject of interest for many chemists and biologists working worldwide.^15,16^

Over the years, 1,3,4-thiadiazoles have been playing a crucial role in inhibiting tyrosine kinases, and their impact is noteworthy.^17,18^ Understanding the effect of thiadiazole compounds in inhibiting them has helped in gaining deeper insights for advancing their synthetic procedures. The action of various substituents and other functional groups connected to the thiadiazole ring is better understood with molecular docking and observed SAR trends. Tyrosine kinases function by adding phosphate groups to tyrosine residues within proteins, which alter their activity. They play a pivotal role in several cell signaling pathways. When mutations occur, certain tyrosine kinases may remain persistently active, which leads to cancer development or uncontrollable cell proliferation.^19–23^

Vascular endothelial growth factor (VEGF) is a key signaling molecule involved in both vasculogenesis and angiogenesis. It leads to increased microvascular permeability, thereby facilitating endothelial cell division and migration. Many tumors exhibit elevated levels of VEGF due to an imbalance between proangiogenic and anti-angiogenic molecules. There are three subtypes, namely, VEGFR-1 (Fms-like tyrosine kinase 1), VEGFR-2 (fetal liver kinase I) and VEGFR-3 (Fms-like tyrosine kinase receptor 4 or vasculotropin receptor 3). Predicting the selectivity of kinase inhibitors based on their chemical structure and sequence is challenging. In VEGFR-2, kinase I inhibitors engage with the ATP-binding site through one to three hydrogen bonds that resemble those formed by ATP. Conversely, type II inhibitors indirectly compete with ATP by occupying a hydrophobic pocket next to the ATP binding site. While some type II inhibitors can establish hydrogen bonds with the ATP binding site, this interaction is not an essential factor in determining their effectiveness.^24–27^

Epidermal growth factor receptors (EGFRs), also known as ErbB1 and HER1, are a type of tyrosine kinase. The inhibition of EGFR kinase activity is regarded as a promising approach for cancer therapy. EGFR inhibitors are a group of medications employed to manage major prevalent cancers, such as lung, breast, colorectal and pancreatic cancers. EGFR is a main mediator in cell signaling pathways, which include key processes such as cell proliferation, apoptosis, angiogenesis and metastatic spread of cells. Thus, by inhibiting EGFR, it is convenient to block cell proliferation in cancer cells, which could later lead to the formation of deadly malignant tumors.^28–31^

The SHP2 protein, a crucial mediator in tumor development, survival and immune evasion (RAS/MAPK/ERK signaling), is blocked by SHP2 inhibitors, a family of targeted anticancer agents. These medications include SHP099, RMC-4550 and TNO155, which bind to SHP2 allosterically, trapping it in an inactive conformation. The use of traditional inhibitors that target the PTP binding site, like PHPS1, GS-493, and NSC-87877, is typically used as the main strategy to reduce the catalytic activity of SHP2. However, poor bioavailability and low cell permeability are common problems associated with conventional inhibitors. Therefore, there have been several attempts in cancer research to develop molecules that can overcome the above-mentioned issues.^32–35^

1,3,4-Thiadiazole compounds have been prominently applied in diverse biological fields, and several reviews have been published in recent years, which emphasize the broader biological potential of these scaffolds.^15,36^ This review highlights active thiadiazole-containing heterocyclic compounds with prominent anticancer efficacy that play a crucial role in inhibiting tyrosine kinases, like VEGFR-2 and EGFR, and phosphatases, like SHP2. The literature was gathered using different search databases such as Scopus, Google Scholar, Web of Science, PubMed, and NCBI. The selection of literature studies (from 2021) was done based on the emerging 1,3,4-thiadiazole scaffolds showing good anticancer activity based on the different evaluation techniques and assays related to EGFR, VEGFR-2 and SHP2 inhibition. Their synthetic methods were discussed based on their inhibition criteria. Therefore, this review is very helpful for chemists and biologists who work on oncogenic research for developing small molecules such as thiadiazole hybrids. Their inhibition, selectivity index, molecular docking results, and SAR observations play key roles in determining their prominent activity and action.

## 2. Synthetic developments of 1,3,4-thiadiazole hybrids

### 2.1 VEGFR-2 kinase inhibitors

Elkady *et al.* constructed a new thiadiazole molecule 7 showing VEGFR-2 inhibition and better anticancer efficacy. The synthesis commenced with the preparation of 4-acetamidoacetophenone 3 from 4-aminoacteophenone 1 and acetic anhydride 2, followed by treatment with methyl hydrazinecarbodithioate 4 to give a Schiff's base intermediate 5. Finally, a hydrazonoyl chloride derivative 6 was reacted with this intermediate 5 to afford the target compound 7. It displayed strong VEGFR-2 enzyme inhibition tendency with an IC_50_ value of 40.65 ± 3.37 nM, which is a better IC_50_ value than that of the standard reference drug sorafenib, provided such comparison is applicable with respect to the original study. Its superiority is also observed with good cytotoxicity, with an IC_50_ value of 6.13 μM against MCF-7 human breast cancer cells. The selectivity index (SI) of 5.86 showed the compound's ability to target MCF-7 breast cancer cells. Moreover, the molecular docking results revealed the presence of hydrogen bonding, with Asp1046 and Glu885 residues oriented towards the VEGFR-2 active site. The target compound was docked with the protein (PDB ID: 4ASD) using the Discovery Studio 4.0 software (Scheme 1).^37^ Likewise, Elgammal *et al.* designed a thiadiazole compound 11 with a methyl piperidinyl ring as a hydrophobic tail and a 4-anisyl group as a hydrophobic head occupying the allosteric site of the VEGFR-2 target, with its synthesis starting from the formation of Schiff's base derivative 9 from compound 8, followed by a subsequent reaction with linker 10. It displayed VEGFR-2 enzyme inhibition with an IC_50_ value of 58.13 ± 2.99 nM. It exhibited strong cytotoxicity against MCF-7 and MDA-MB-231 breast cancer cells with IC_50_ values of 15.83 and 9.66 μM, respectively, when compared with the standard drug sorafenib. Also, it displayed minimum toxicity towards normal WI-38 cells with selectivity indices of 4.50 (WI-38/MCF-7) and 7.38 (WI-38/MDA-MB-231). Thus, compound 11 was found to be more selective towards MDA-MB-231 than MCF-7 cancer cells. Furthermore, three H-bonds with the Asp1044, Cys917 and Cys1043 amino acid residues within the active site of VEGFR-2 favoured strong interactions in compound 11. The target compound was docked with the protein (PDB ID: 2OH4) using the GROMACS 2021 tool (Scheme 2).^38^

**Scheme 1:**
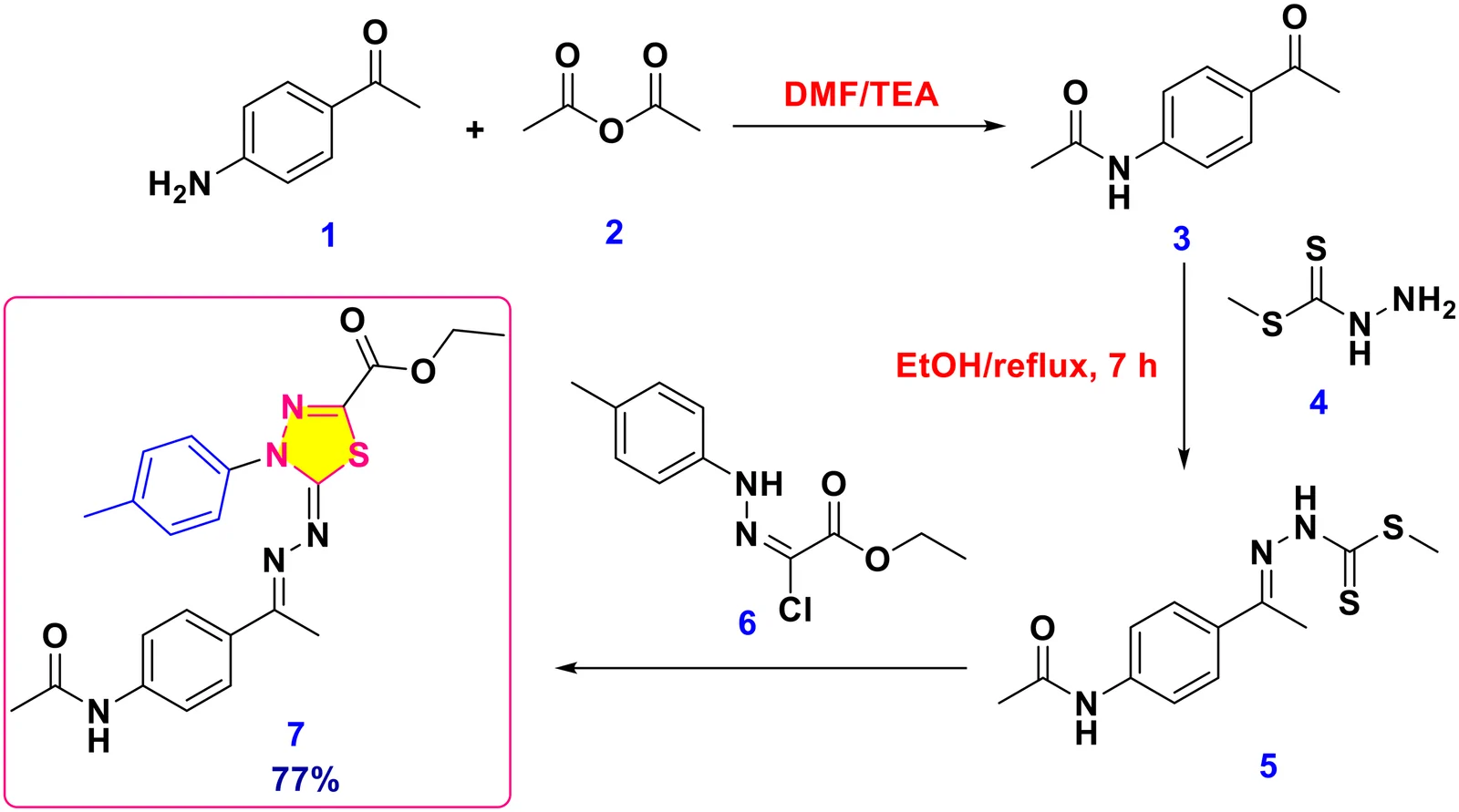
Synthesis of the thiadiazole-linked hydrazonoyl acetamide VEGFR-2 inhibitor 7.

**Scheme 2:**
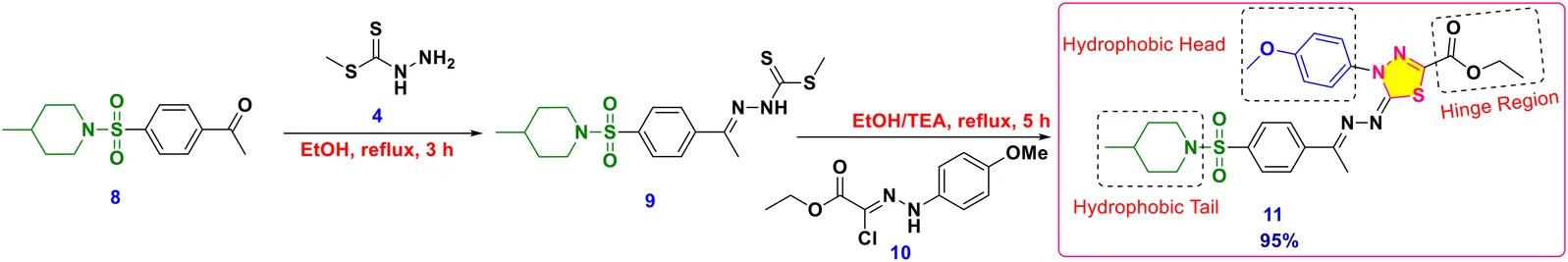
Synthesis of the thiadiazole-linked hydrazonoyl sulfonamide analogue 11.

Metwaly *et al.* designed a synthetic route for nicotinamide-dihydrothiadiazole 16, which was later found to be a good candidate compound with anticancer efficacy against MDA-MB-231 cancer cells with promising VEGFR-2 inhibition. The synthesis involved the reaction of 4-aminoacetophenone 1 and nicotinoyl chloride 12 to give a condensed product 13, which subsequently formed a Schiff's base 14 upon treatment with methyl hydrazinecarbodithioate 4. The final product 16 was obtained in good yield upon reacting 14 with hydrazone 15. It showed IC_50_ values of 6.92 ± 0.4 μM against MDA-MB-231 cells and 0.098 ± 0.05 μM against VEGFR-2, which were comparable to those of the standard drug sorafenib. The selectivity index was found to be 8.68 (WI-38/MDA-MB-231), reflecting minimal toxicity towards the normal cell line WI-38 and displaying its therapeutic efficacy. Two H-bonds with Asp1046 and Glu885 were found *via* its ethylidenehydrazine moiety, and the important hydrophobic thiadiazole carboxylate part was found to have good interaction with Leu1019, Leu889, Ile892, and Ile888. Additionally, compound 18 was synthesized by reacting intermediate 14 with 17, which exhibited potent anticancer efficacy with IC_50_ values of 4.64 ± 0.3 μM against MDA-MB-231 cells and 0.095 ± 0.05 μM against VEGFR-2. A selectivity index of 15.86 for MDA-MB-231 was found when tested with normal cells, while the selectivity index of sorafenib was lower compared to that of compound 18. The final compound was docked with the protein (PDB ID: 4ASD) using the GROMACS 2021 tool (Scheme 3).^39,40^

**Scheme 3:**
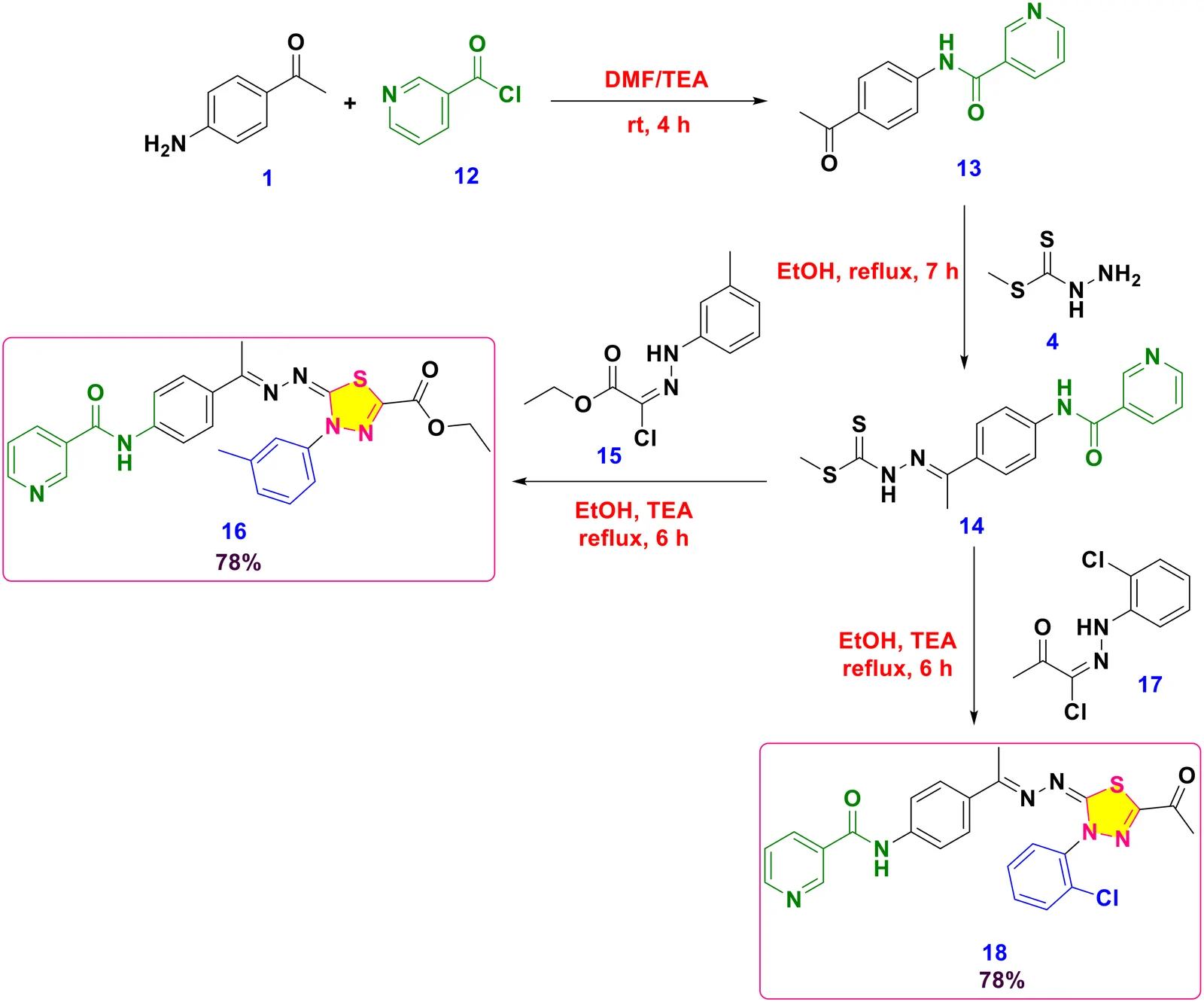
Synthesis of nicotinamide-dihydrothiadiazole derivatives 16 and 18 with VEGFR-2 inhibition.

Alsfouk and coworkers prepared a thiadiazole-benzenesulfonamide hybrid 21 from the precursor 19*via* an acid-catalyzed condensation reaction under standard reflux conditions for 7 h to give the intermediate 20, whose treatment with a hydrazonoyl chloride derivative 17 resulted in compound 21. Compound 21 showed potent anticancer efficacy against HepG2 cancer cells with an IC_50_ value of 17.89 μM, closely associated with that of the standard drug sorafenib. Interestingly, compound 21 exhibited potent inhibition against VEGFR-2 and B-Raf with IC_50_ values of 0.15 and 0.11 μM, respectively. Two H-bonds with Asp1044 and Leu838 effectively bound the compound within the VEGFR-2 pocket, and three H-bonds with Lys483, Phe595, and Asp594 bound it within the active site of B-Raf. The hydrophobic thiadiazole core part effectively enhanced the activity along with the 2-chloro substituent. The docking results were obtained when the final compound was docked with the protein (PDB ID: 2OH4) using the Discovery Studio 4.0 software (Scheme 4).^41^

**Scheme 4:**
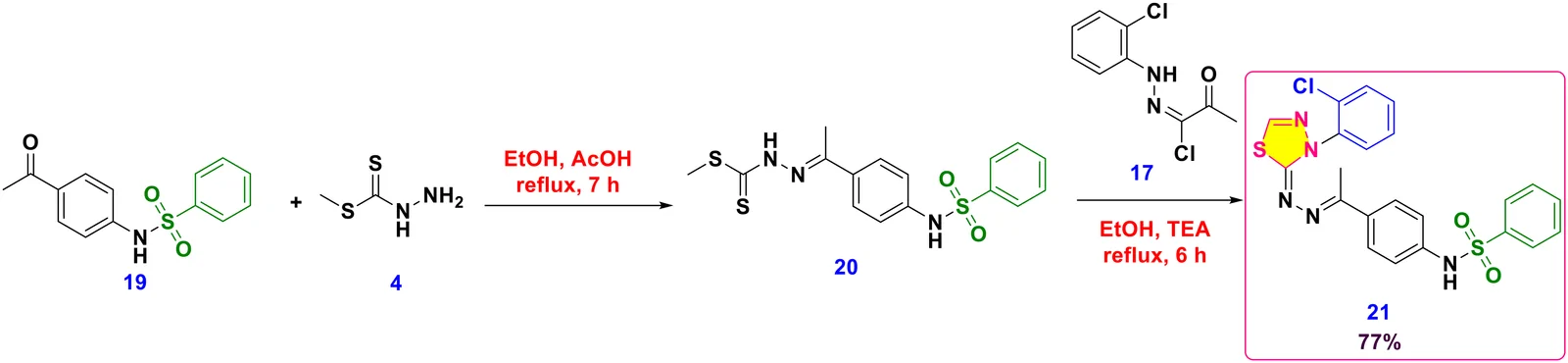
Synthesis of the thiadiazole-benzenesulfonamide hybrid 21 (dual VEGFR-2/B-Raf inhibitor).

Eissa and coworkers reported several thiadiazole compounds accomplished *via* the same synthetic routes as discussed above. Compound 22 was effective against MCF-7 and HepG2 cancer cells with IC_50_ values of 0.04 and 0.18 μM, respectively. Additionally, it exhibited greater inhibition against VEGFR-2 with an IC_50_ value in the nanomolar range (103 nM).^42^ Likewise, compound 23 was active against the MCF-7 and HepG2 cancer cell lines with IC_50_ values of 0.05 and 0.14 μM, respectively. Notably, it surpassed the performance of sorafenib in terms of the activity displayed against VEGFR-2 with an IC_50_ value of 0.024 μM. Selectivity indices of 3.8 and 1.4 were found against MCF-7 and HepG2, respectively, after taking results from normal WI-38 cells.^43^ Compound 24 displayed promising action against HepG2 cancer cells with an IC_50_ value of 0.17 ± 04 μM, close to that of sorafenib. Furthermore, it exhibited good inhibition against VEGFR-2 with an IC_50_ value of 0.283 μM.^44^ In contrast, compound 25 exhibited potent activity without having a substituent on the phenyl ring, acting as the hydrophobic part of the lead compound by itself. It possessed good cytotoxicity with IC_50_ values of 5.69 μM against MDA-MB-231 cancer cells and 0.083 μM against VEGFR-2 kinase, showing inhibitory activity superior to that of the standard drug sorafenib. Also, its selectivity index of 9.05 when tested with the WI-38 normal cells indicated its therapeutic potential.^45^ Also, these four compounds (22–25) exhibited favourable drug-likeness, high safety margin and low mutagenicity, attributed to their selective chemical reactivity and high chemical stability. The docking poses showed several interactions when docked with the protein (PDB ID: 2OH4) using the MOE2014 software (Fig. 1). The compounds discussed thus far are listed with their comparative VEGFR-2 kinase inhibition activity in Table 1.

**Fig. 1:**
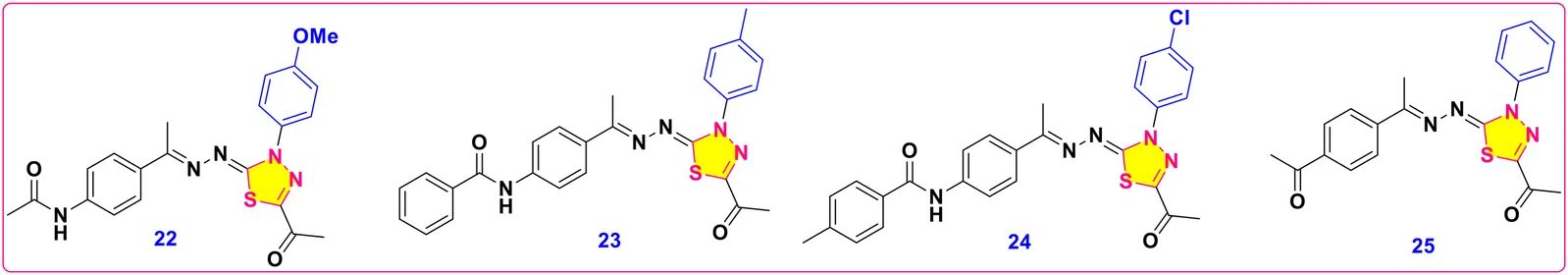
Pharmacologically active thiadiazole-containing VEGFR-2 kinase inhibitors 22–25.

**Table 1:** Overview and comparison of the VEGFR-2 kinase inhibition activity of different thiadiazole-containing heterocyclic hybrids

Compound	Substituent	Major interactions	Observed SAR trends	VEGFR-2: IC_50_ value	Reference VEGFR-2 inhibitor	Cancer cell line	Limitations	Reference
7	4-Tolyl group attached to the thiadiazole moiety	H-Bonding with the Asp1046 and Glu885 residues of the VEGFR-2 active site	Electron-donating methyl group improved the activity	40.65 ± 3.37 nM	Sorafenib: 53.32 ± 2.52 nM	MCF-7: 6.13 μM	Does not establish a broad kinase selectivity profile; does not demonstrate tumour-growth suppression	37
11	4-Methoxy group attached to the thiadiazole moiety	Three H-bonds with Asp1044, Cys917 and Cys1043 within the active site of VEGFR-2	A strong electron-donating methoxy group favoured electronic interaction, contributing to increased cytotoxicity	58.13 ± 2.99 nM	Sorafenib: 53.32 ± 2.52 nM	MCF-7: 15.83 μM; MDA-MB-231: 9.66 μM	Moderate cellular potency relative to the reference drug	38
16	3-Tolyl group attached to the thiadiazole carboxylate moiety	Interaction with Leu1019, Leu889, Ile892, and Ile888 within the active site of VEGFR-2	Electron-donating methyl group at the 3rd position of the phenyl ring improved the activity	0.098 ± 0.05 μM	Sorafenib: 0.10 ± 0.05 μM	MDA-MB-231: 6.92 ± 0.4 μM	Requires further pharmacokinetic optimisation	39
18	2-Chloro group attached to the thiadiazole moiety	Two H-bonds with Asp1046 and Glu885 bound within the pocket of the VEGFR-2 active portion	A hydrophobic 2-Cl substituent improved the cytotoxicity when compared with hydrophilic substituents like 4-NO_2_	0.095 ± 0.05 μM	Sorafenib: 0.10 ± 0.05 μM	MDA-MB-231: 4.64 ± 0.3 μM	Animal studies are required for the assessment of anti-tumour efficacy	40
21	2-Chloro group attached to the thiadiazole moiety	Two H-bonds with Asp1044 and Leu838 effectively bound the compound within the VEGFR-2 pocket and three H-bonds with Lys483, Phe595, and Asp594 within the active site of B-Raf	Hydrophobic 2-Cl substituent improved the cytotoxicity	0.15 μM	Sorafenib: 0.10 μM	HepG2: 17.89 μM	No *in vivo* efficacy validation reported; cellular mechanism requires further validation with respect to cell cycle arrest, apoptosis, and caspase-3 activation	41
22	4-Methoxy moiety attached to the thiadiazole core structure	Two H-bonds with Glu883 and Asp1044 within the active site pocket of VEGFR-2	Hydrophobic methoxy substituent enhanced the cytotoxicity	103 nM	Sorafenib: 41 nM	MCF-7: 0.04 μM; HepG2: 0.18 μM	Computational ADMET assessment identifies low predicted aqueous solubility as a concern	42
23	4-Tolyl group linked to the thiadiazole active pharmacophore	Two crucial H-bonds with Glu883 and Asp1044 within the active site pocket of VEGFR-2	The hydrophobic methyl (in 4-tolyl) group was responsible for increased anticancer efficacy	0.024 μM	Sorafenib: 0.041 μM	MCF-7: 0.05 μM; HepG2: 0.14 μM	Early-stage evidence is achieved which requires further experimental validation	43
24	4-Chlorophenyl group attached to the thiadiazole moiety	Two crucial H-bonds with Glu883 and Asp1044 within the active site pocket of VEGFR-2	The pharmacophore region along with the 4-chloro substituent enhanced the activity due to its hydrophobic nature and stabilizing ability	0.283 μM	Sorafenib: 0.041 μM	HepG2: 0.17 ± 04 μM	Computational findings require experimental validation	44
25	Phenyl group attached to the thiadiazole moiety	Two crucial H-bonds with Glu883 and Asp1044 within the active site pocket of VEGFR-2	The phenyl group having rich aromaticity and hydrophobic character induced better cytotoxicity	0.083 μM	Sorafenib: 0.100 μM	MDA-MB-231: 5.69 μM	Safety evidence remains preliminary and requires further studies and experimentation	45

Toolabi and coworkers synthesized the phenylacetamide-derived thiadiazole derivative 34 and tested its anticancer efficacy and VEGFR-2 inhibition tendency. The synthesis started with the treatment of a thiosemicarbazide 26 with carbon disulfide 27 to form the thiadiazole core structure 28, followed by its *N*-acetylation using acetic anhydride in acetic acid and subsequent condensation with 4-nitrochlorobenzene 30 to give a nitro intermediate 31, whose reduction to amine 32 was followed by treatment with 4-chlorophenylisocyanate 33 to give the target compound 34. The similarity in the phenylacetamide moiety in the target compound 34 as well as sorafenib accounts for the better design and construction of such derivatives. Compound 34 showed prominent results with an IC_50_ value of 9.23 μM against A431 cancer cells when compared with sorafenib and had no cytotoxicity over normal HDF cells. Western blot analysis revealed the blocking ability of compound 34 towards the phosphorylation of VEGFR-2 in A431 cells. It showed an IC_50_ value of 7.77 μM against VEGFR-2. The thiadiazole ring occupied the hydrophobic region, established several π-alkyl interactions with Val848, Ala866, and Leu1035 and polar interactions involving H-bonds with Glu885, Cys919, and Asp1046 and stabilized the ATP binding site. These results were obtained when the final compound was docked with the protein (PDB ID: 2OH4) using the Discovery Studio 4.0 tool (Scheme 5).^46^

**Scheme 5:**
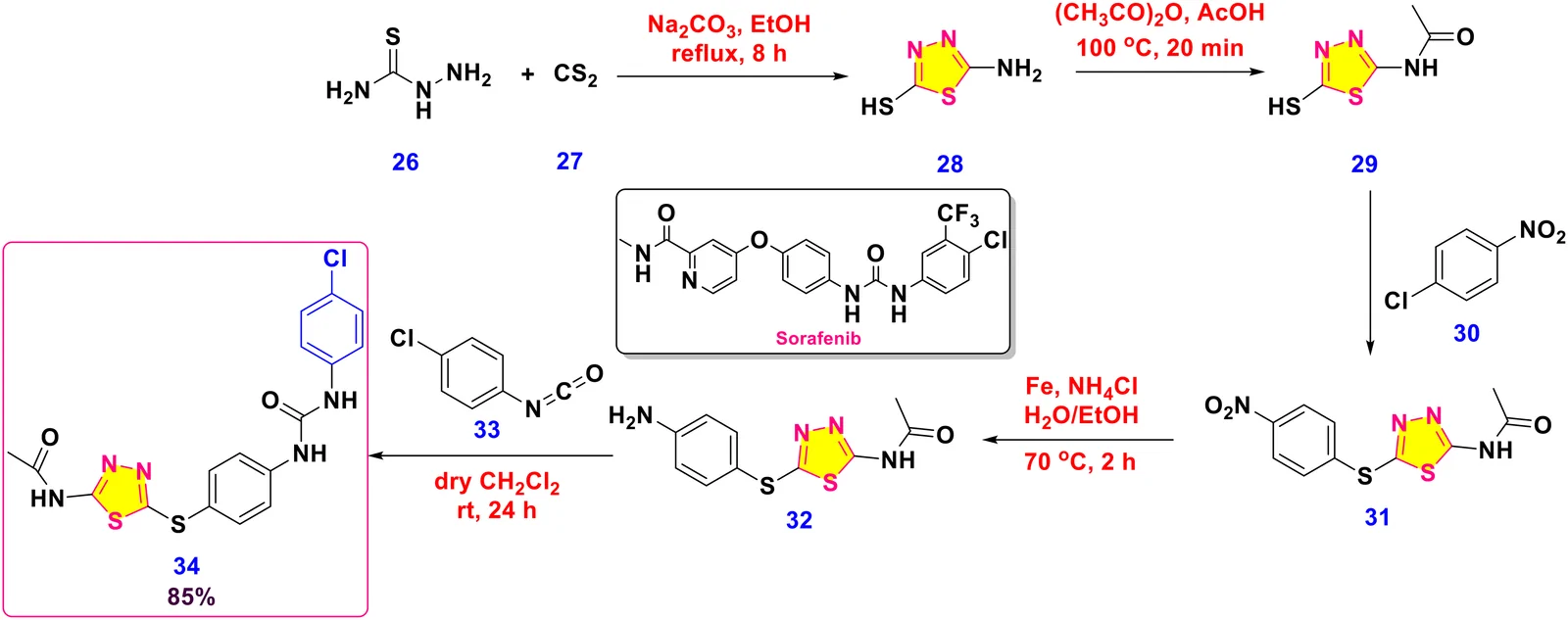
Synthesis of the phenylacetamide-derived thiadiazole derivative 34 for VEGFR-2 inhibition.

Atta-Allah and other workers used a 2-furyl-substituted thiadiazole derivative 40 with promising anticancer effect against VEGFR-2 kinase with an IC_50_ value of 9.4 nM compared with the standard drug pazopanib. Cytotoxicity studies revealed the potential antiproliferative capacity of compound 40 in four different cell lines, namely, HepG2, MCF-7, HCT-116, and PC3, with IC_50_ values (μM) of 6.8 ± 0.65, 4.36 ± 2.0, 5.26 ± 2.85, and 8.01 ± 2.1, respectively. These results were closer to the results displayed by the standard drug doxorubicin. Also, this compound showed a strong DNA-binding affinity of 36.06 μM. The synthesis involved a green microwave-assisted organic pathway for the conversion of compound 37 to 40*via* a 2-step reaction. Prior to this, normal heating was performed to produce the important intermediate 37, which was later irradiated with microwaves to give 38 and finally reacted with furfural to give the target compound 40. The thiadiazole linked-2-furyl group formed H-bonds with Glu885 or Asp1046 within the active site of the VEGFR-2 binding pocket. The thiophene portion engaged in a hydrophobic interaction with Lys868. These interactions were obtained when docking was done with the protein (PDB ID: 3VNT) using the MOE2020 tool (Scheme 6).^47^

**Scheme 6:**
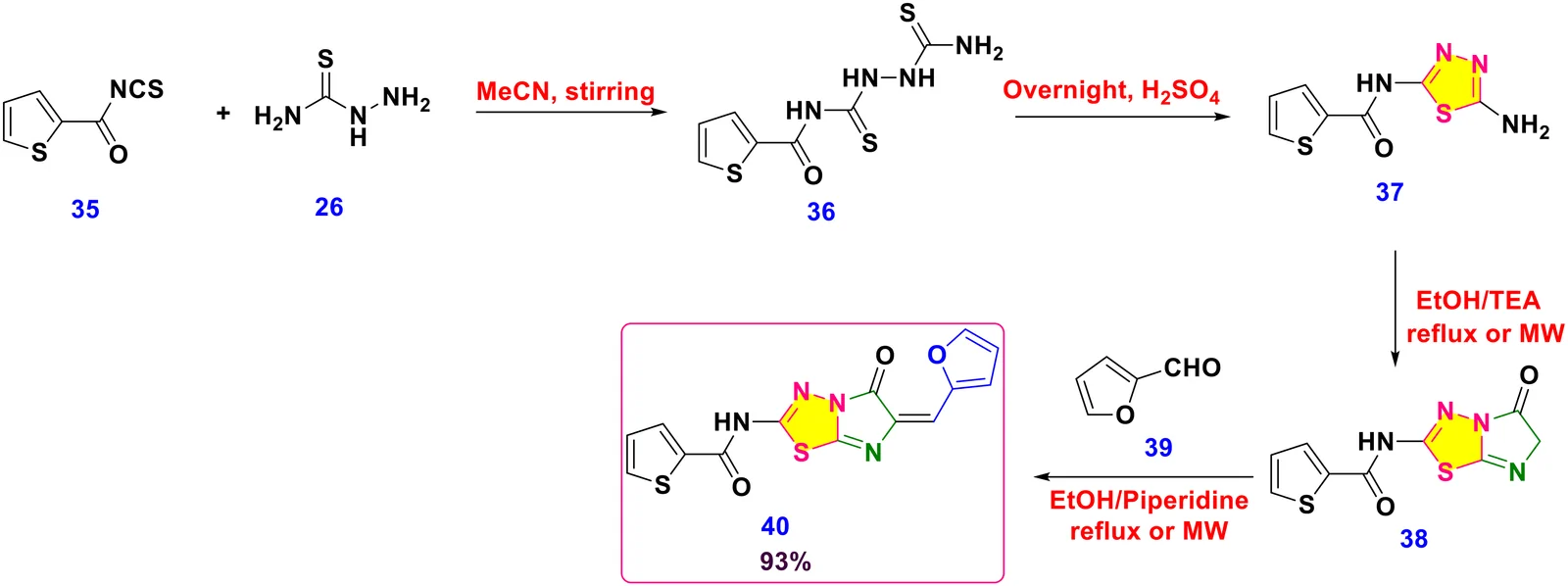
Synthesis of the 2-furyl-derived thiadiazole hybrid 40 with VEGFR-2 kinase inhibition activity.

As seen above, a microwave-assisted method was used for producing furan-2-derived thiadiazole 47 with good cytotoxic and antiproliferative effects against colon and breast cancer cells. The synthetic route involved four steps: (i) the conversion of 2-furanacetylchloride 41 to 2-furanacetylisothiocyanate 42 using ammonium isothiocyanate; (ii) its subsequent condensation with 2-cyanoacetohydrazide 43 to give 44; (iii) the treatment of 44 with glacial acetic acid under microwave irradiation to produce 45; and (iv) the reaction of 45 with lactic acid 46, leading to the formation of the final compound 47. It was active against HCT-116 cells with an IC_50_ value of 8.65 ± 3.7 μM in comparison with doxorubicin. Additionally, it was effective in inhibiting VEGFR-2 kinase with an IC_50_ value of 7.4 nM compared with pazopanib. The selectivity index was found to be 9.63, showing a positive character with respect to its therapeutic potential. Molecular docking studies showed hydrophobic interactions with Val848, Ala866, Leu840, Leu1035 and Cys919 fitting within the pocket of the VEGFR-2 active site. Molecular docking was done with the protein (PDB ID: 4ASE) using the AutoDock software (Scheme 7).^48^

**Scheme 7:**
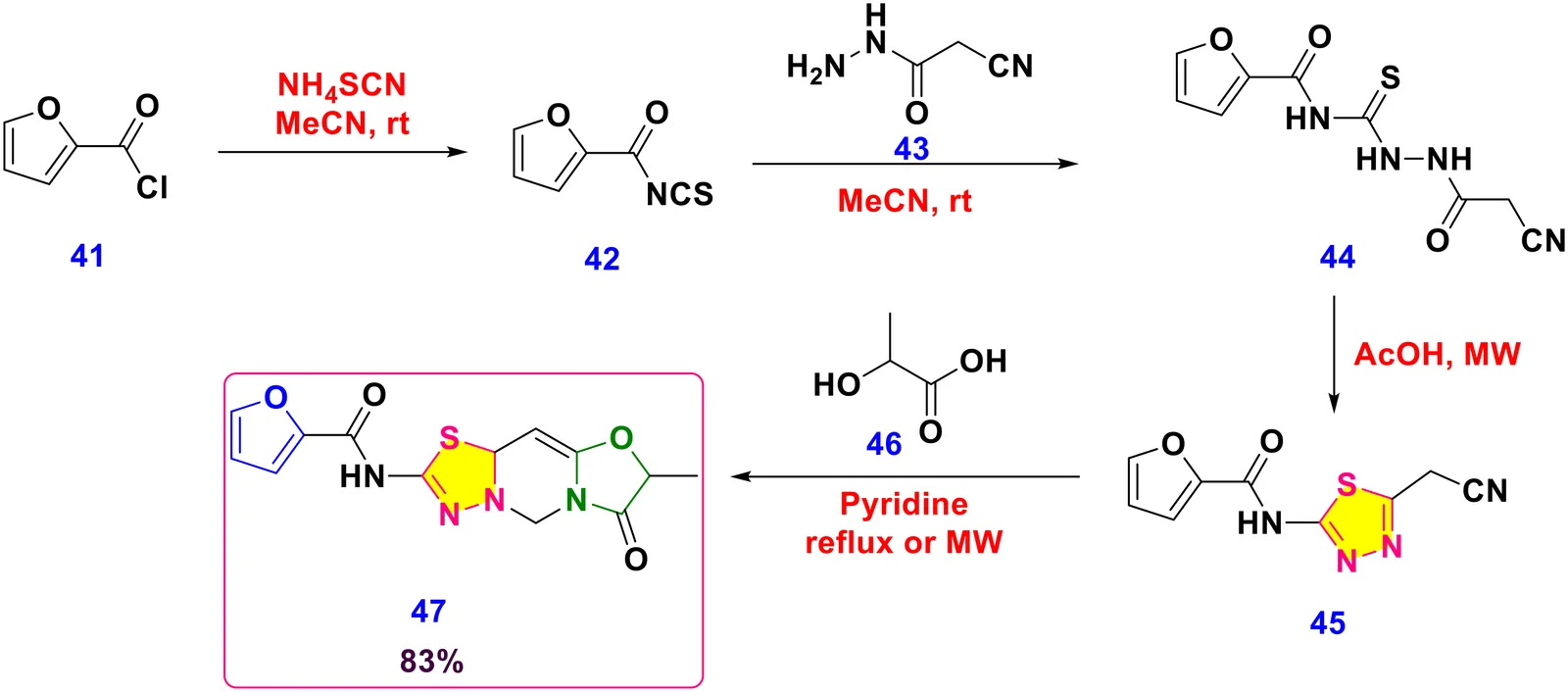
Synthesis of the furan-2-derived thiadiazole derivative 47 with VEGFR-2 kinase inhibitory activity.

Masoudinia constructed a quinazoline-linked thiadiazole hybrid 54 with good anticancer efficacy against MCF-7 cancer cells. The synthesis started with the cyclization of 2-aminobenzoic acid 48 to quinazoline-4(3*H*)-one 49, whose nitration introduced a nitro group 50 and chlorination using thionyl chloride led to the formation of the substrate 51 for the synthesis of the target compound 54 from 53. 53 was produced by the usual procedure of constructing 5-amino-1,3,4-thiadiazole-2-thiol 28, followed by its reaction with 4-methylphenylisocyanate 52. Finally, the condensation of 51 and 53 led to the formation of the final compound 54. It possessed good cytotoxicity and anticancer activity with an IC_50_ value of 25 ± 0.001 μM against MCF-7 human breast cancer cells compared with the standard drug etoposide. Its IC_50_ value against VEGFR-2 was observed to be 25.8 ± 0.0008 μM. Furthermore, it possessed promising inhibition of the VEGFR-2 kinase. Additionally, it did not violate any of the five rules of Lipinski, indicating its preliminary potential for oral bioavailability in the future. Molecular docking showed that the polar interactions involving H-bonds with Glu885, Asp1046, Cys919 and Lys868 stabilized the ATP binding site. The target compound was docked with the protein (PDB ID: 2OH4) using the GROMACS 2020 software (Scheme 8).^49^

**Scheme 8:**
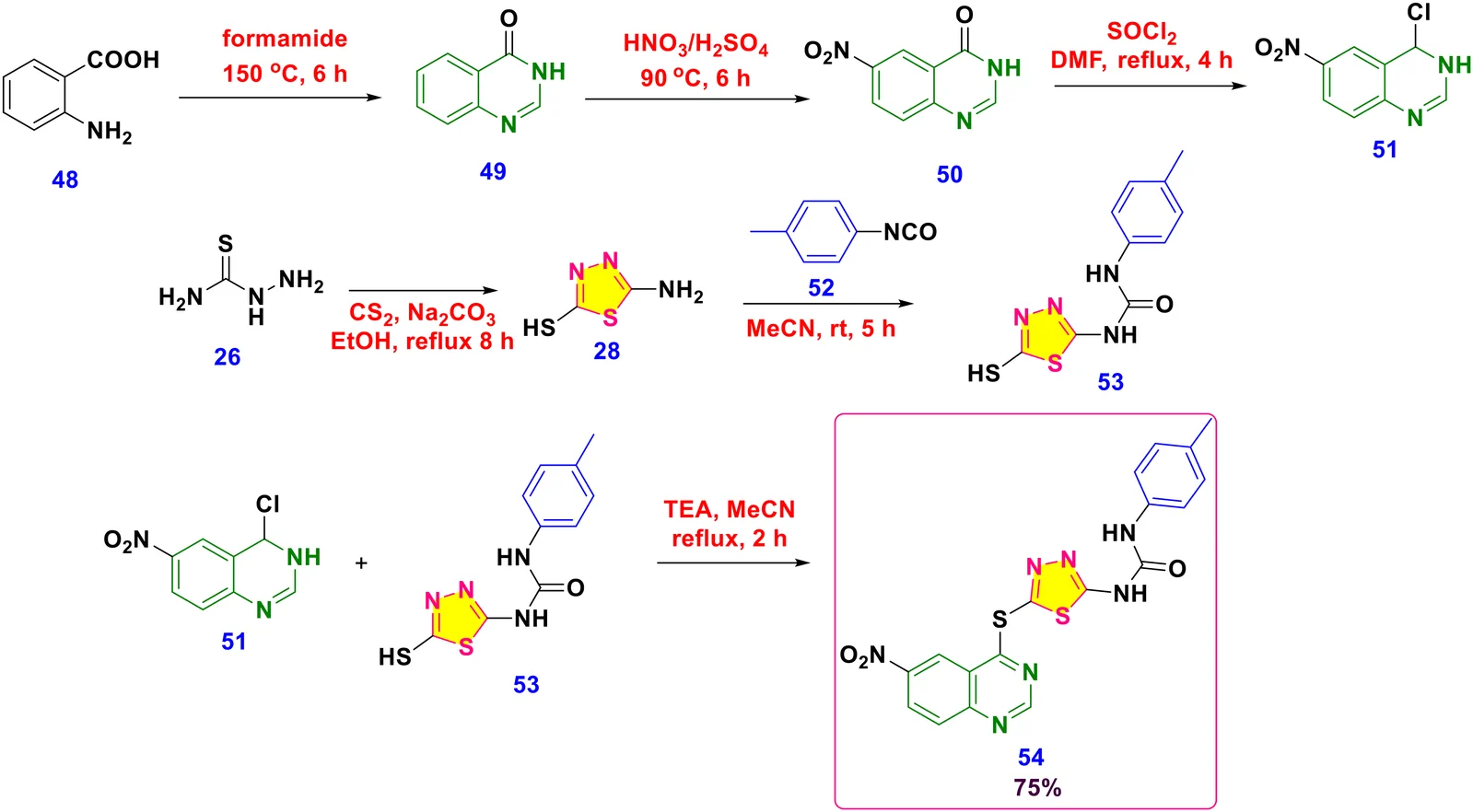
Synthesis of the quinazoline-tethered thiadiazole hybrid 54 with anticancer efficacy.

Ewes *et al.* designed a benzothiazole-derived thiadiazole derivative 60 with notable anticancer effects against HCT-116 colorectal cancer cells with an IC_50_ value of 6.21 ± 0.4 μM compared with that of sorafenib. Additionally, it played a dual role in inhibiting both VEGFR-2 and BRAF with IC_50_ values of 0.071 and 0.194 μM, respectively. However, its selectivity index of 6.6 with normal WI-38 cells unlocked the preliminary door to its therapeutic application. Compound 60 was prepared *via* two routes. The first route involved the preparation of the *N*-substituted chloroacetamide derivative of benzothiazole 57 from 2-aminobenzothiazole 55 and chloroacetyl chloride 56. In the second route, 59 was prepared by the usual steps of forming 28 and reacting it with 58. Finally, by combining the two substrates 57 and 59, the target compound 60 was obtained. Molecular docking studies showed several H-bonds with Cys532, Trp531, Thr529, Val471, Leu514, Ala481, and Phe583. The target compound was docked with the protein (PDB ID: 4ASD) using the Discovery Studio 4.0 software (Scheme 9).^50^

**Scheme 9:**
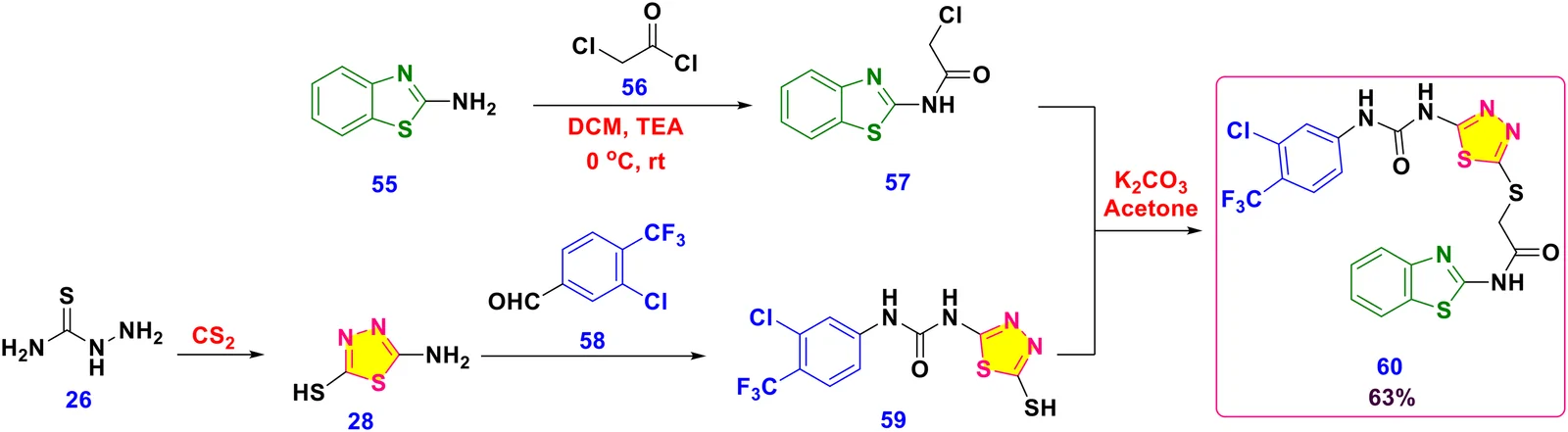
Synthesis of the benzothiazole-derived thiadiazole derivative 60 with dual VEGFR-2 and BRAF inhibition.

An overview of the thiadiazole-containing heterocyclic molecules with VEGFR-2 kinase inhibition activity is provided in Table 2.

**Table 2:** Overview and comparison of the VEGFR-2 kinase inhibition activity of different thiadiazole-containing heterocyclic hybrids

Compound	Substituent	Major interactions	Observed SAR trends	VEGFR-2: IC_50_ value	Reference VEGFR-2 inhibitor	Cancer cell line	Limitations	Reference
34	4-Chlorophenyl connected to a urea type scaffold of thiadiazole	Polar interaction involving H-bonds with Glu885, Cys919, and Asp1046 stabilized the ATP binding site	Hydrophobic 4-Cl substituent improved the cytotoxicity	7.77 μM	Sorafenib: 10.42 μM	A431: 9.23 μM	Limited cancer-cell line panel and requires further diverse experimentation across different cell lines	46
		π-Alkyl interactions with Val848, Ala866, and Leu1035 by the thiadiazole core moiety						
40	2-Furyl substituent attached to the thiadiazole moiety	The thiadiazole linked-2-furyl group forms H-bonds with Glu885 or Asp1046 within the active site of the VEGFR-2 binding pocket. The thiophene portion formed a hydrophobic interaction with Lys868	2-Furyl and thiophene groups increased the cytotoxicity along with the thiadiazole core region	9.4 nM	Pazopanib: 9.7 nM	HepG2: 6.8 ± 0.65 μM; MCF-7: 4.36 ± 2.0 μM; HCT-116: 5.26 ± 2.85 μM; and PC3: 8.01 ± 2.1 μM	Annexin-V testing of compounds 4b and 9a showed no substantial increase in early or late apoptotic cells under the reported conditions	47
47	2-Furylacetamide substituent linked to the thiadiazole moiety and cyclized oxazole ring	Hydrophobic interactions with Val848, Ala866, Leu840, Leu1035 and Cys919 fitting within the pocket of the VEGFR-2 active site	Active vinylic group of the oxazole ring showed increased cytotoxicity	7.4 nM	Pazopanib: 9.7 nM	HCT-116: 8.65 ± 3.7 μM	No *in vivo* validation was reported	48
54	4-Tolyl group attached to the thiazole urea moiety	Polar interactions involving H-bonds with Glu885, Asp1046, Cys919 and Lys868 stabilize the ATP binding site	Electron-donating 4-methyl group increased the anticancer efficacy	25.8 ± 0.0008 μM	Etoposide: 29.8 ± 0.0007	MCF-7: 25 ± 0.001 μM	Reported *in silico* ADME predictions require further experimental validation	49
60	3-Chloro-4-trifluoromethylphenyl group attached to the urea moiety of thiadiazole	H-bonding with Cys532, Trp531, Thr529, Val471, Leu514, Ala481, and Phe583	Hydrophobic 3-chloro-4-trifluoromethyl phenyl tail enhanced the cytotoxicity in association with the thiadiazole core region	0.071 μM	Sorafenib: 0.069 μM	HCT-116: 6.21 ± 0.4 μM	Further structural modifications and optimization are necessary to enhance the VEGFR-2 inhibitory efficacy	50

### 2.2 EGFR kinase inhibitors

Kurban and coworkers designed a synthetic route for the synthesis of the pyrazole-thiadiazole hybrid 69 with superior activity against the A549 cell line and EGFR inhibitory activity. The synthesis involved two phases, including the simultaneous formation of pyrazole and thiadiazole. The pyrazole derivative was prepared using acetophenone 61 and 4-nitrobenzaldehyde 62 to give a chalcone intermediate 63, which reacted with hydrazine hydrate to form a pyrazoline moiety 64, and its treatment with chloroacetyl chloride 56 resulted in the formation of *N*-acetyl pyrazoline 65. On the other hand, phenylisothiocyanate (Edman's reagent) 66 was reacted with a hydrazine hydrate to give 67, whose subsequent reaction with CS_2_/NaOH led to the formation of the thiadiazole core molecule 68, and combining it with the previously synthesized *N*-acetyl pyrazoline 65 furnished the final compound 69. It showed potent activity against A549 cancer cells with an IC_50_ value of 1.537 ± 0.097 μM when compared with doxorubicin and inhibited EGFR enzyme inhibition with an IC_50_ value of 0.024 ± 0.002 μM in comparison to erlotinib. Molecular docking showed cation–π interaction with Lys721 and H-bonds with Asp831, Cys773 and Met769. These interactions were the result of docking with the protein (PDB ID: 1M17) using the Schrodinger Maestro software (Scheme 10).^51^

**Scheme 10:**
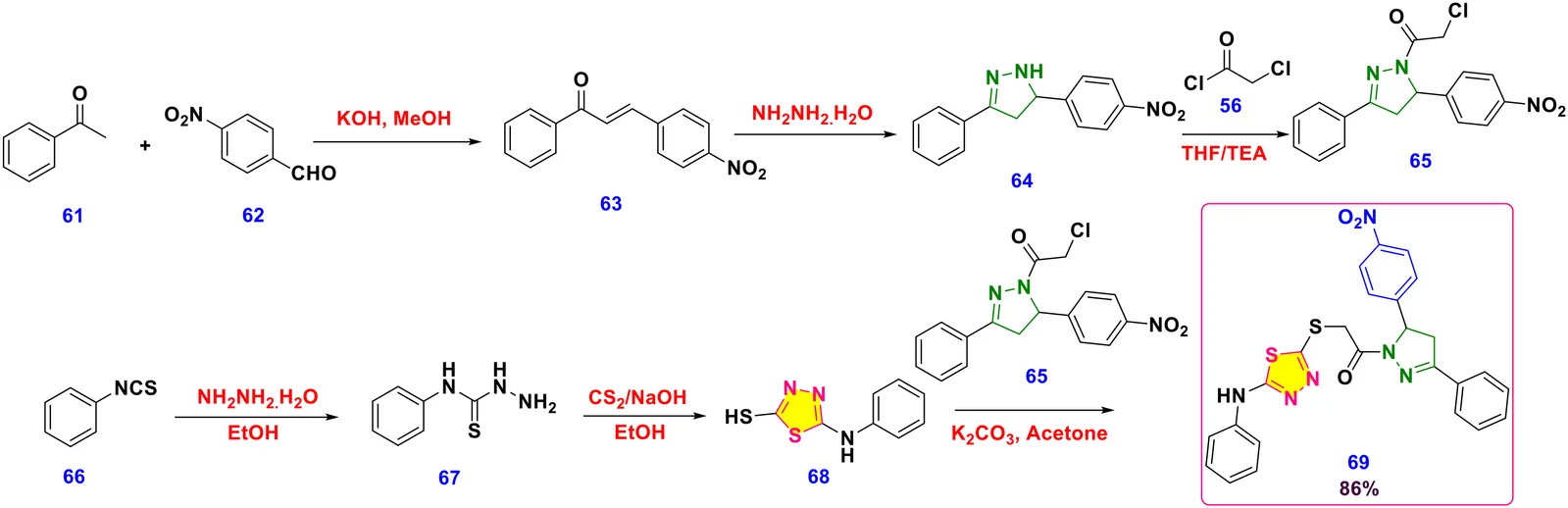
Synthesis of the pyrazole-thiadiazole analogue 69 with EGFR inhibition activity.

Serag *et al.* produced tris-thiadiazole 74, which exhibited excellent anticancer efficacy against MCF-7 human breast cancer cells and EGFR inhibition. The synthesis started from the usual generation of the thiadiazole core molecule 28 and its reaction with benzyl chloride 70 as the linker spacer. The amino group 71 was then acetylated using chloroacetyl chloride 56 to give 72. Finally, reacting it with 1,3,4-thiadiazol-2,5-dithiol 73 generated compound 74. It possessed good anticancer effects with an IC_50_ value of 3.31 ± 0.1 μM compared with gefitinib. Moreover, it showed promising EGFR inhibition with an IC_50_ value of 0.08 μM. The selectivity index of this compound when tested with the normal WI-38 cells was found to be 13.29. Therefore, its therapeutic effects make it a potential candidate for the development of tris-thiadiazole-based drug molecules. Molecular docking showed arene-cation interaction with Lys745 and Val726, and the thiadiazole moiety formed H-bonds with Leu718, Pro794, His805, Thr790, Thr854, and Leu844. The target compound was docked with the protein (PDB ID: 4WKQ) using the MOE2020 software (Scheme 11).^52^

**Scheme 11:**
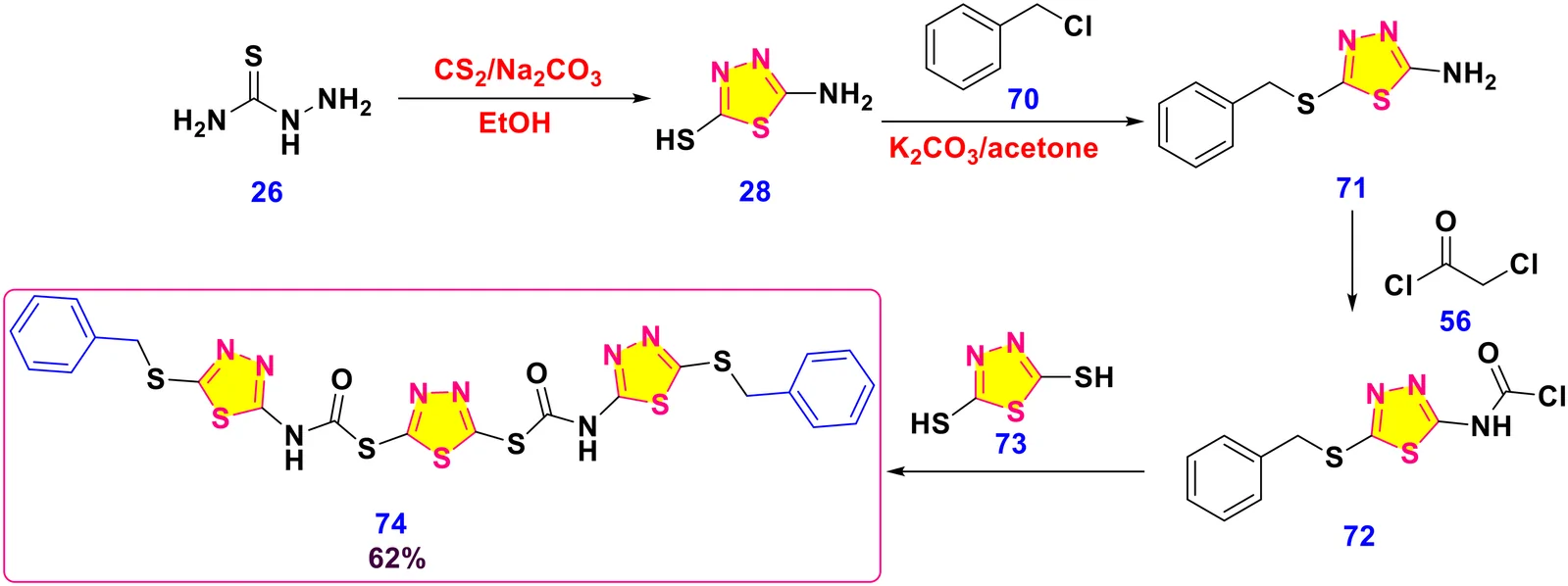
Synthesis of the EGFR inhibitor tris-thiadiazole 74.

Abdulkhaleq *et al.* produced a morpholine-derived thiadiazole scaffold 81 exhibiting good cytotoxicity and antiproliferative effects with EGFR inhibition displaying an IC_50_ value of 11 μM compared with the standard drug gefitinib. However, the synthesis procedure involved reactions between 3-hydroxymethyl benzoate 75 and benzyl bromide 76 to give the condensed product 77, which upon treatment with hydrazine hydrate and CS_2_/KOH, produced the thiadiazole scaffold 79. The morpholine-containing alkyl bromide 80 when reacted with 79, produced the lead molecule 81. The molecular docking results displayed arene-cation interaction within hydrophobic pockets containing residues like Cys775, Thr854, Leu788, Lys745, Met793, Asp855 and Leu844. These interactions were obtained by docking the target compound with the protein (PDB ID: 4WKQ) using the AutoDock software (Scheme 12).^53^

**Scheme 12:**
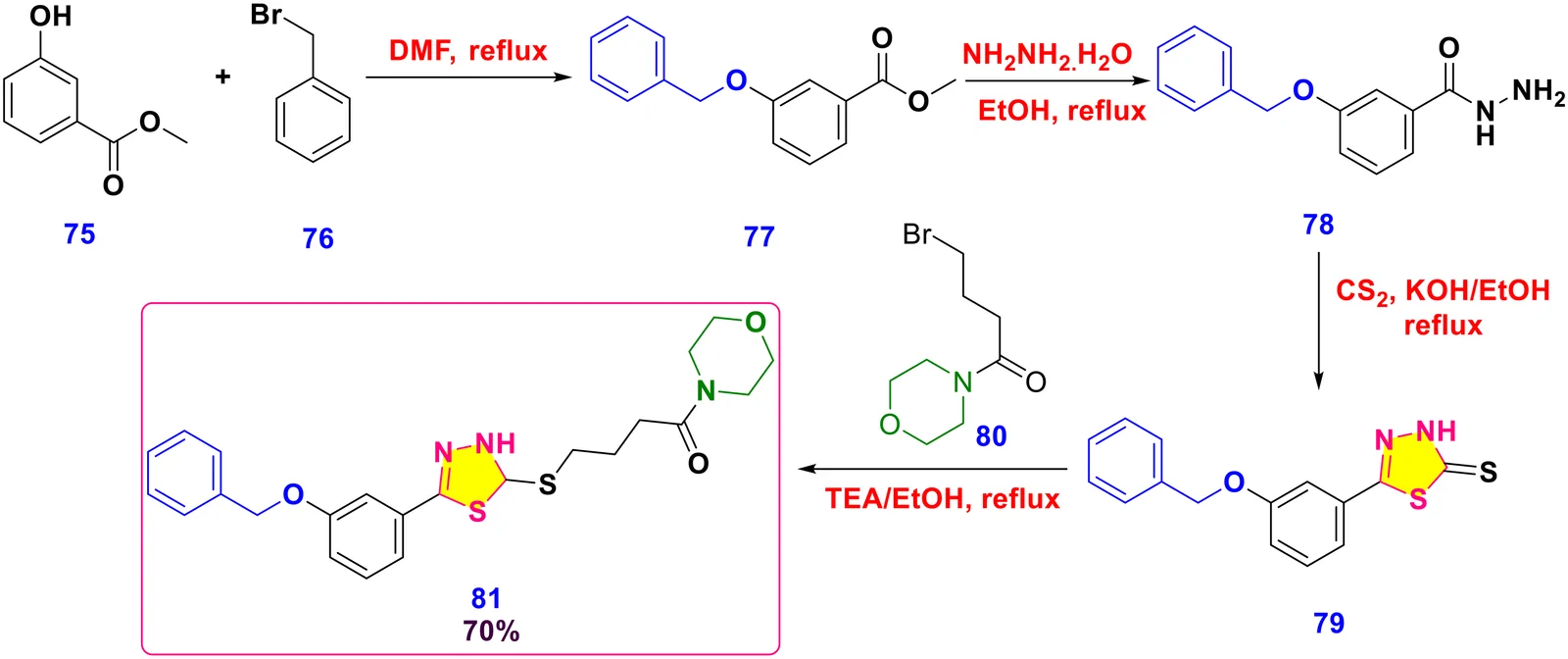
Synthesis of the morpholine-containing thiadiazole EGFR inhibitor 81.

Arzu *et al.* constructed thiadiazole analogues with a benzenesulfamoyl moiety 85, which displayed promising activity in terms of anticancer efficacy with EGFR inhibition. The synthesis followed the normal route of constructing 28, followed by its condensation with 4-fluorobenzoyl chloride 82 under freezing conditions using triethylamine as the base for the reaction. The intermediate 83 was then treated with 2-chloro-*N*-(4-sulfamoylphenyl)acetamide 84 to give the lead compound 85. It showed good cytotoxicity and antiproliferative efficacy against MCF-7 cancer cells with an IC_50_ value of 2.884 ± 0.124 μM when compared with doxorubicin. The EGFR inhibitory activity was observed with an IC_50_ value of 0.028 ± 0.0012 μM when compared with erlotinib. Additionally, this compound was found to be effective in inhibiting the COX-2 enzyme. Therefore, it acts as a dual EGFR/COX-2 inhibitor with potency for anticancer as well as anti-inflammatory activities. Furthermore, molecular docking showed H-bond interactions with Lys704, Lys721, Thr766, Met769, Arg817, Asn818, Thr830, and Asp831 residues lying within the binding pocket of EGFR. The final compound was docked with the protein (PDB ID: 1M17) using the Schrodinger Maestro software (Scheme 13).^54^

**Scheme 13:**
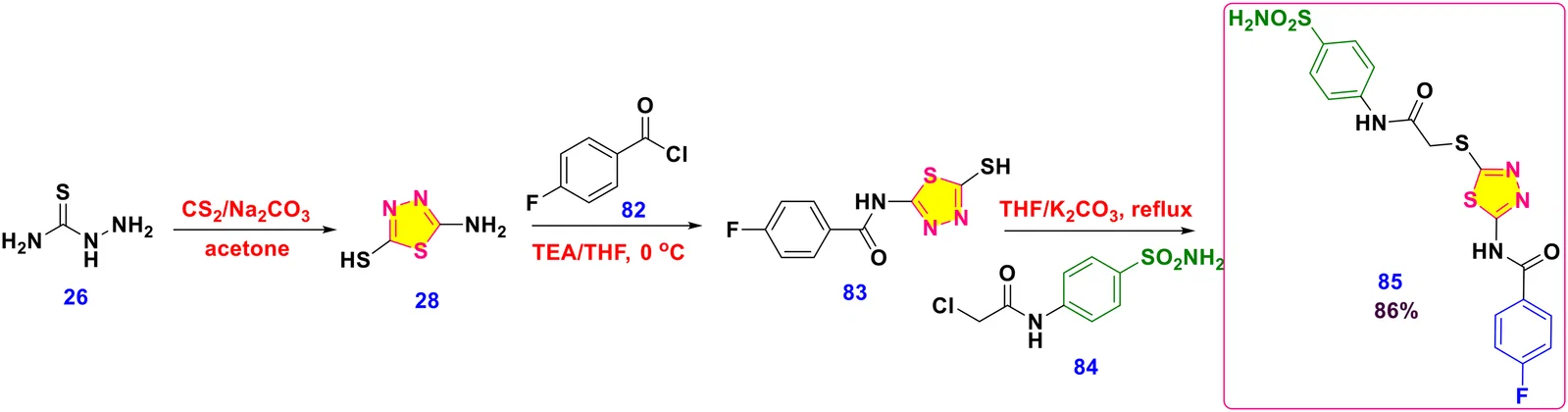
Synthesis of the benzenesulfamoyl-derived thiadiazole 85, a dual EGFR/COX-2 inhibitor.

Ismail *et al.* designed a phenethyl-linked thiadiazole-tethered benzimidazole scaffold 94 with EGFR inhibitory activity. The synthetic route followed sequential steps involving the reaction of *o*-phenylenediamine 86 with 4-methoxybenzaldehyde 87 to give a benzimidazole core 88 moiety, which was then treated with ethyl chloroacetate 89 to give an *N*-ester 90; the subsequent treatment of 90 with hydrazine hydrate and phenethyl isothiocyanate 92 gave 93, which furnished the cyclized product 94 upon treatment with 1 N NaOH. Compound 94 inhibited up to 68% of EGFR kinase activity, attributed to various molecular interactions found to be within the binding pocket of the target enzyme. Also, it tends to obey Lipinski's rule of five, making it compatible for the preliminary screening of oral bioavailability. Additionally, molecular docking results displayed one H-bond interaction with Lys721 and two H-bonds with Asp831 in the EGFR active site. The final compound was docked with the protein (PDB ID: 1M17) using the Schrodinger Maestro software (Scheme 14).^55^

**Scheme 14:**
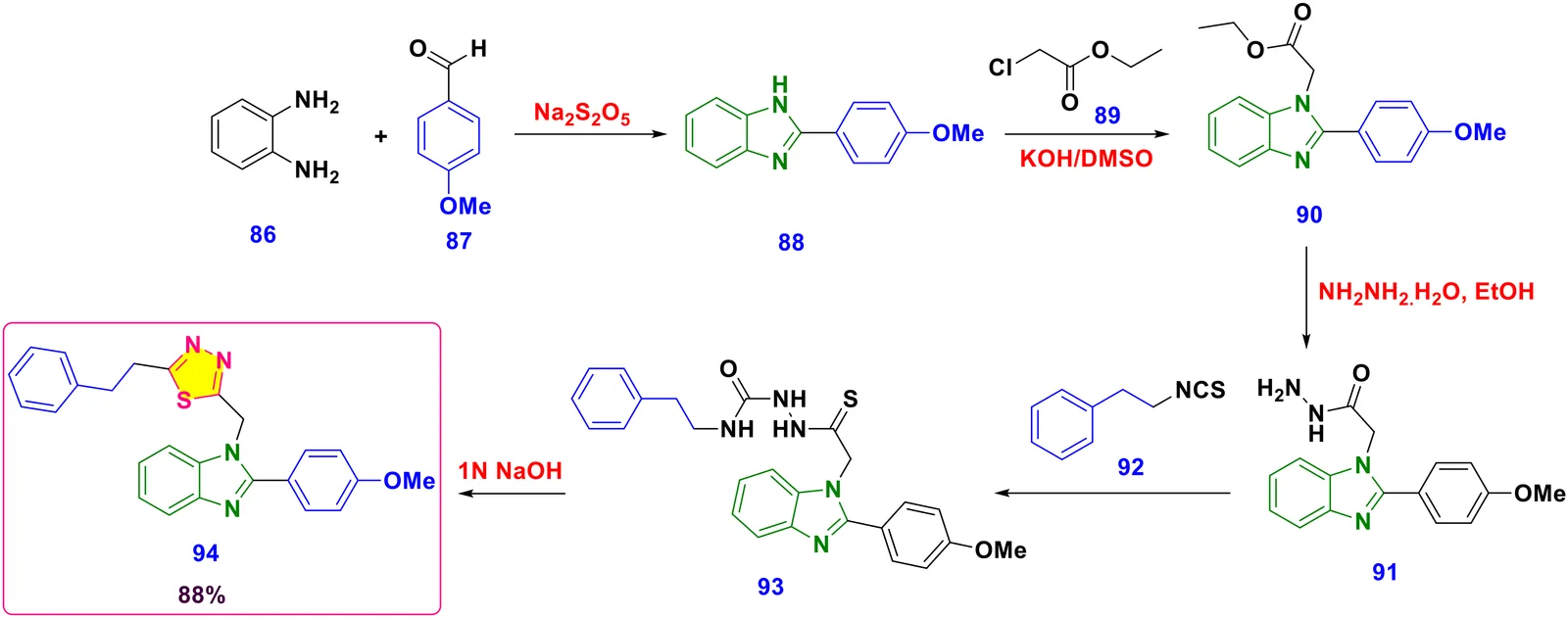
Synthesis of the phenethyl-linked thiadiazole-tethered benzimidazole scaffold 94.

Cai and coworkers synthesized the thiadiazole-containing acrylamide moiety 98 by reacting 2-(2-bromophenyl)acetic acid 95 with thiosemicarbazide 26 to obtain thiadiazole 96, which was subsequently reacted with acryloyl chloride 97 using NaOH under freezing conditions. Compound 98 showed effective antiproliferative activity with an IC_50_ value of 25.84 ± 4.73 μM against the EGFR-expressed A431 cell line when compared with that of the standard drug gefitinib. Molecular docking results showed interactions such as H-bonds with Cys775, Thr854 and Leu338 within the binding pocket of EGFR. The target compound was docked with the protein (PDB ID: 1M17) using the AutoDock software (Scheme 15).^56^

**Scheme 15:**
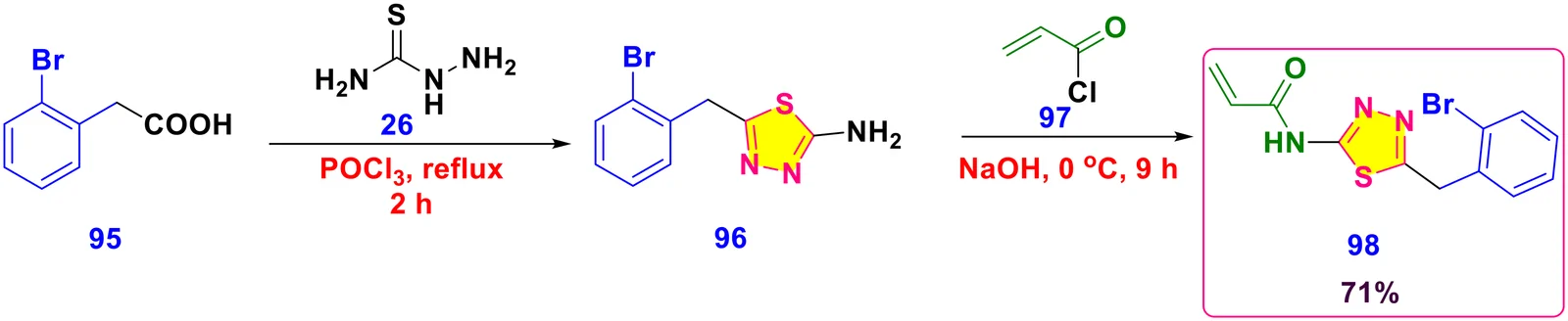
Synthesis of the EGFR inhibitor thiadiazole-containing acrylamide 98.

Thorat *et al.* constructed 2-amino-1,3,4-thiadiazoles containing an oxidibenzene moiety 102 from 3-phenoxybenzaldehyde 99 and thiosemicarbazide 26 in a photocatalytic reactor using a blue LED lamp of 460 nm and 2,4,6-tri(*p*-tolyl)pyrylium tetrafluoroborate (TPP-TFB) as the organocatalyst for 16 h. The obtained intermediate 100 was treated with 4-bromoaniline 101 using an XPhos Pd G1 catalyst for 6 h to give the lead molecule 102, which showed promising activity against the EGFR-expressed MCF-7 cell line with an IC_50_ value of 0.59 ± 0.19 μM and a selectivity index of 26.31. Molecular docking showed H-bonds with Asp831 and Glu738, enhancing the stabilization with the protein (PDB ID: 1M17) using the Schrodinger Maestro tool (Scheme 16).^57^

**Scheme 16:**
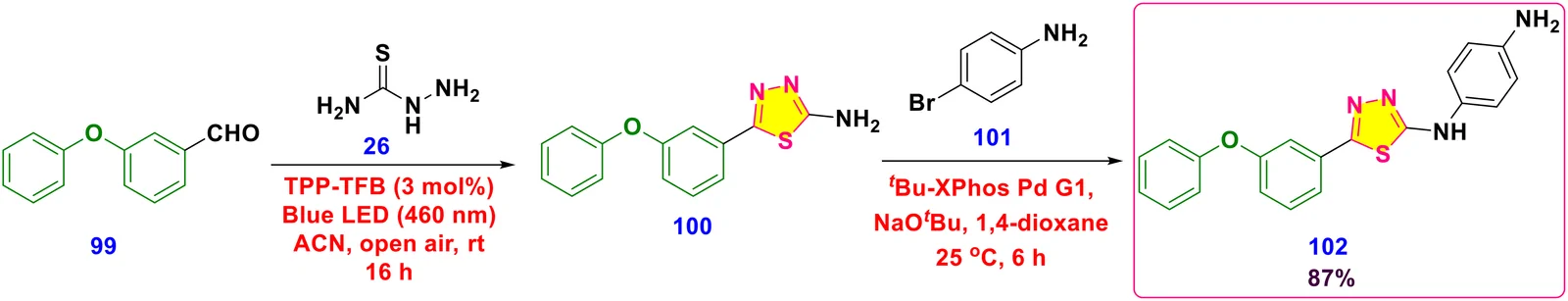
Photocatalytic synthesis of 2-amino-1,3,4-thiadiazoles 102.

An overview of thiadiazole-containing heterocyclic molecules with EGFR kinase inhibitory activity is provided in Table 3.

**Table 3:** Overview and comparison of the EGFR kinase inhibition activity of different thiadiazole-containing heterocyclic hybrids

Compound	Substituent	Major interactions	Observed SAR trends	EGFR: IC_50_ value/% inhibition	Reference EGFR inhibitor	Cancer cell line	Limitations	Reference
69	Phenyl ring attached to the amino group of the thiadiazole moiety	Cation–π interaction with Lys721. H-bond with Asp831, Cys773 and Met769	The phenyl ring, itself having rich aromaticity and hydrophobic character, induced better cytotoxicity	0.024 ± 0.002 μM	Erlotinib: 0.002 ± 0.001 μM	A549: 1.537 ± 0.097 μM	Molecular dynamics (MD) simulation analyses indicated that the phenyl ring had a flexible conformation	51
74	Benzyl thiol linker at the terminal ends of the thiadiazole molecule	Arene-cation interaction with Lys745 and Val726; H-bonds of the thiadiazole moiety formed H-bonds with Leu718, Pro794, His805, Thr790, Thr854, and Leu844	Benzyl thiol group having good hydrophobic character possessed a tendency for increasing the cytotoxic efficacy	0.08 μM	Gefitinib: 0.04 μM	HCT-116: 3.31 ± 0.1 μM	Animal control experiments are required for further validation	52
81	Morpholinone alkyl side chain and benzyloxy group attached to the phenyl ring of the thiadiazole	Arene-cation interaction with hydrophobic pockets containing residues like Cys775, Thr854, Leu788, Lys745, Met793, Asp855 and Leu844	The benzyloxy group, being electron-donating in nature, improved the cytotoxic performance of the unsubstituted morpholine-containing thiadiazole analogue	11 μM	Gefitinib: 20.8 μM	A549: 27 μM	Further *in vivo* studies are required	53
85	4-Fluorophenyl substituent linked to thiadiazole through an acetamide moiety	H-bonds with Lys704, Lys721, Thr766, Met769, Arg817, Asn818, Thr830, and Asp831 residues lying within the binding pocket of EGFR	An electron-withdrawing fluoro group tended to increase the anticancer effect along with the contribution of the benzenesulfamoyl group	0.028 ± 0.0012 μM	Erlotinib: 0.002 ± 0.0001 μM	MCF-7: 2.884 ± 0.124 μM	Lack of superior cytotoxicity against A549 cancer cells	54
94	4-Anisyl group of benzimidazole and phenethyl moiety attached to thiadiazole	One H-bond with Lys721 and two H-bonds with Asp831 in the EGFR active site	The electron-donating 4-anisyl group tended to increase the cytotoxicity offered by thiadiazole and benzimidazole	68% inhibition	Erlotinib: 96% inhibition	—	Limited cancer-cell line panel and requires further diverse experimentation across different cell lines	55
98	2-Bromobenzyl substituent linked to the thiadiazole ring	H-bonds with Cys775, Thr854 and Leu338 within the binding pocket of EGFR	Electron-withdrawing fluoro substituent improved the cytotoxicity along with the vinyl group of acrylamides	25.84 ± 4.73 μM	Gefitinib: 26.07 ± 1.12 μM	—	—	56
102	5-Oxidibenzene substituent attached to the thiadiazole ring and -NH_2_ group	H-bonds with Asp831 and Glu738, enhancing the stabilization	An electron-donating amino group stabilized the compound and resulted in enhanced anticancer activity by H-bonding interaction	0.59 ± 0.19 μM	Erlotinib: 0.056 ± 0.06 μM	MCF-7: 2.235 ± 0.012 μM	Exhibited slightly lower absolute biochemical potency against both wild-type and mutated EGFR compared with erlotinib	57

Mostafa and coworkers produced benzothiazole-based imidazothiadiazole compound 106. Benzothiazole-2-carboxylic acid 103 and thiosemicarbazide 26 in POCl_3_ were reacted to give the benzothiazole-thiadiazole amine 104 intermediate, which finally reacted with 4-bromophenacyl bromide 105 to afford 106. Compound 106 showed good results against proliferation in MCF-7 human breast cancer cells and A549 lung cancer cells with IC_50_ values of 8.55 ± 0.73 and 5.89 ± 0.32 μM, respectively, compared with erlotinib. It showed an IC_50_ value of 5.04 ± 0.14 μM against EGFR kinase. Molecular docking showed strong H-bonds with Asp831 and Thr830 lying within the binding pocket of EGFR when docked with the protein (PDB ID: 4HJO) using the AutoDock software (Scheme 17).^58^

**Scheme 17:**
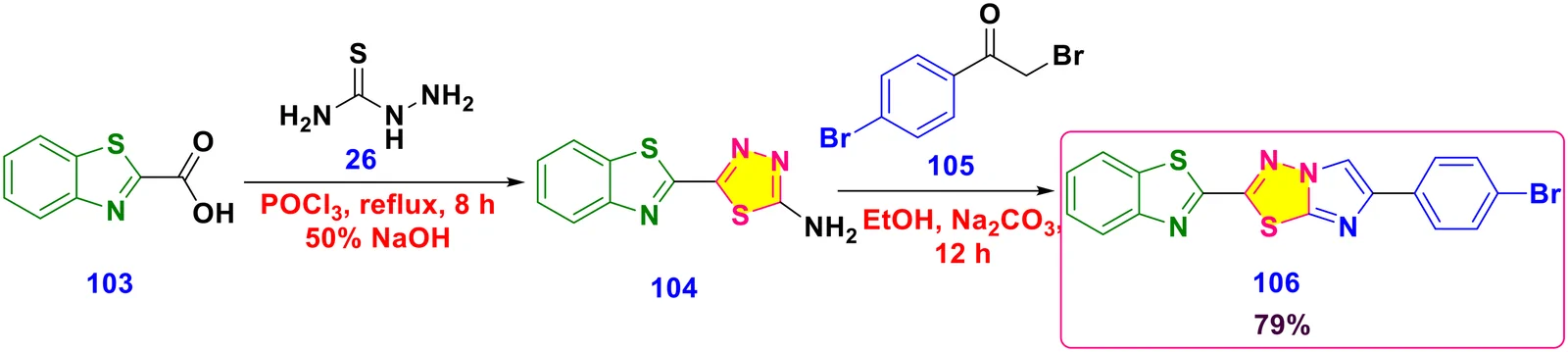
Synthesis of the benzothiazole-based imidazothiadiazole EGFR inhibitor 106.

Ahad *et al.* illustrated a synthetic method for the preparation of a new anticancer lead candidate and EGFR inhibitor 110. The condensation of 28 with isobenzofuran-1,3-dione 107 gave the thiadiazole core 108, which was further reacted with 1,4-diiodobutane 109 in the presence of potassium carbonate and DMF to afford 110. Compound 110 exhibited good inhibitory effects against PC-3 cancer cells with an IC_50_ value of 21.17 ± 7.59 μM in comparison with doxorubicin. It presented a good degree of selectivity against EGFR kinase and therapeutic potential with a selectivity index of 6.4. Molecular docking showed H-bonds with Lys721, Met769 and Val702 within the EGFR active site with the protein (PDB ID: 1M17) using the MOE2014 tool (Scheme 18).^59^

**Scheme 18:**
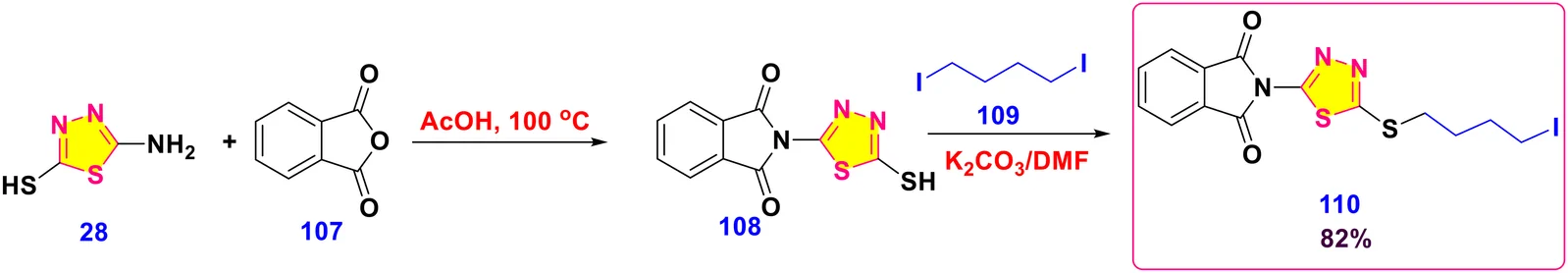
Synthesis of the isoindoline-thiadiazole EGFR inhibitor 110.

Elganzory and coworkers designed thiadiazole-triazole-containing glycoside 117 as an interesting compound showing promising anticancer efficacy against MCF-7 cancer cells. Its synthesis started by reacting 1,2-dibromoethane 112 with the thiadiazole precursor 111 to give 113, and its conversion into the respective azide 114 using sodium azide. This azide 114 was then treated with propargyl glycosides of glucopyranosyl 115 to obtain 116 using a click chemistry approach involving Cu-catalyzed alkyne-azide 1,3-dipolar cycloaddition, and the protected acetyl groups were deprotected using ammonia in methanol at room temperature to give the final compound 117. It showed promising activity with an IC_50_ value of 0.5 μM in comparison with doxorubicin. This action was superior to that of the standard drug, and in terms of its EGFR inhibition, it reflected good action with an IC_50_ value of 0.5 μM compared with erlotinib. Molecular docking revealed interactions such as H-bonds with Met769, Met793 and Met801 within the EGFR binding pocket. The docking poses showed these interactions when the final compound was docked with the protein (PDB ID: 1M17) using the MOE2014 tool (Scheme 19).^60^

**Scheme 19:**
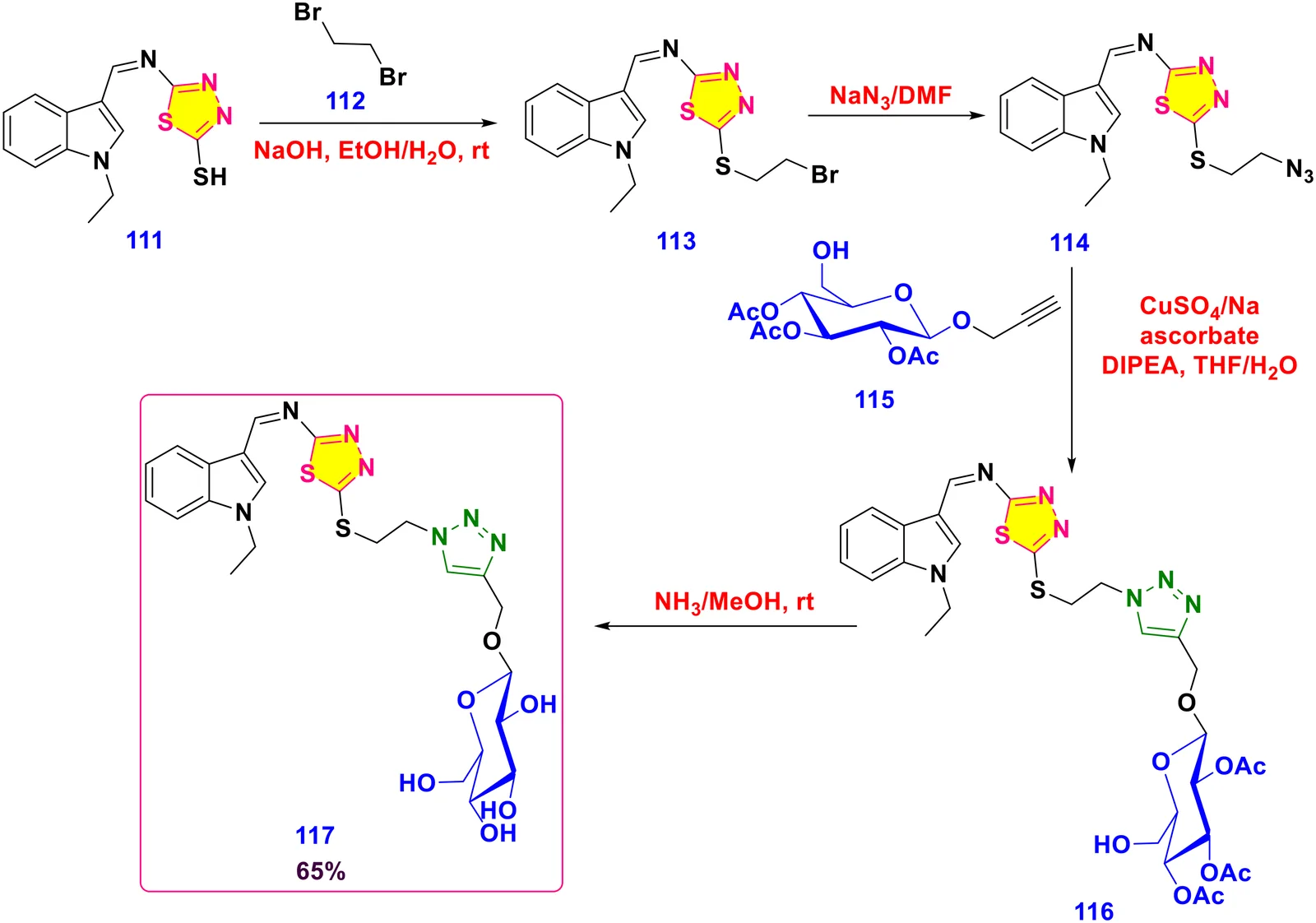
Synthesis of the thiadiazole-triazole-containing glycoside 117.

Li *et al.* designed a long synthetic route for the generation of a quinoline-based thiadiazole hybrid 129 with significant anticancer activity. 2,2-Dimethyl-1,3-dioxane-4,6-dione 118 when treated with trimethyl orthoformate 119 produced 120, and its treatment with 3,4-dimethoxyaniline 121 gave a condensed product 122, which was subsequently treated with phenyl ether to form the quinoline scaffold 123. Methyl-4-(chloromethyl)benzoate 124 was then reacted with the previously formed quinoline 123 to give the quinoline ester 125, which was converted to its respective acid 126 using NaOH and then chlorinated using thionyl chloride 127. The final step involved the reaction between 128 and 127 to give the quinoline-based thiadiazole compound 129. This was effective against A549 and MCF-7 cancer cells with IC_50_ values of 1.03 ± 0.37 and 1.45 ± 0.19 μM, respectively, compared with cabozantinib. The EGFR inhibitory activity was found to be good with an IC_50_ value of 29.30 ± 3.28 μM. Molecular docking showed that the H-bonds with Asp831, Val702 and Thr766 residues were embedded within the EGFR active binding site. The docking poses showed these interactions when the final compound was docked with the protein (PDB ID: 1M17) using the MOE2016 tool (Scheme 20).^61^

**Scheme 20:**
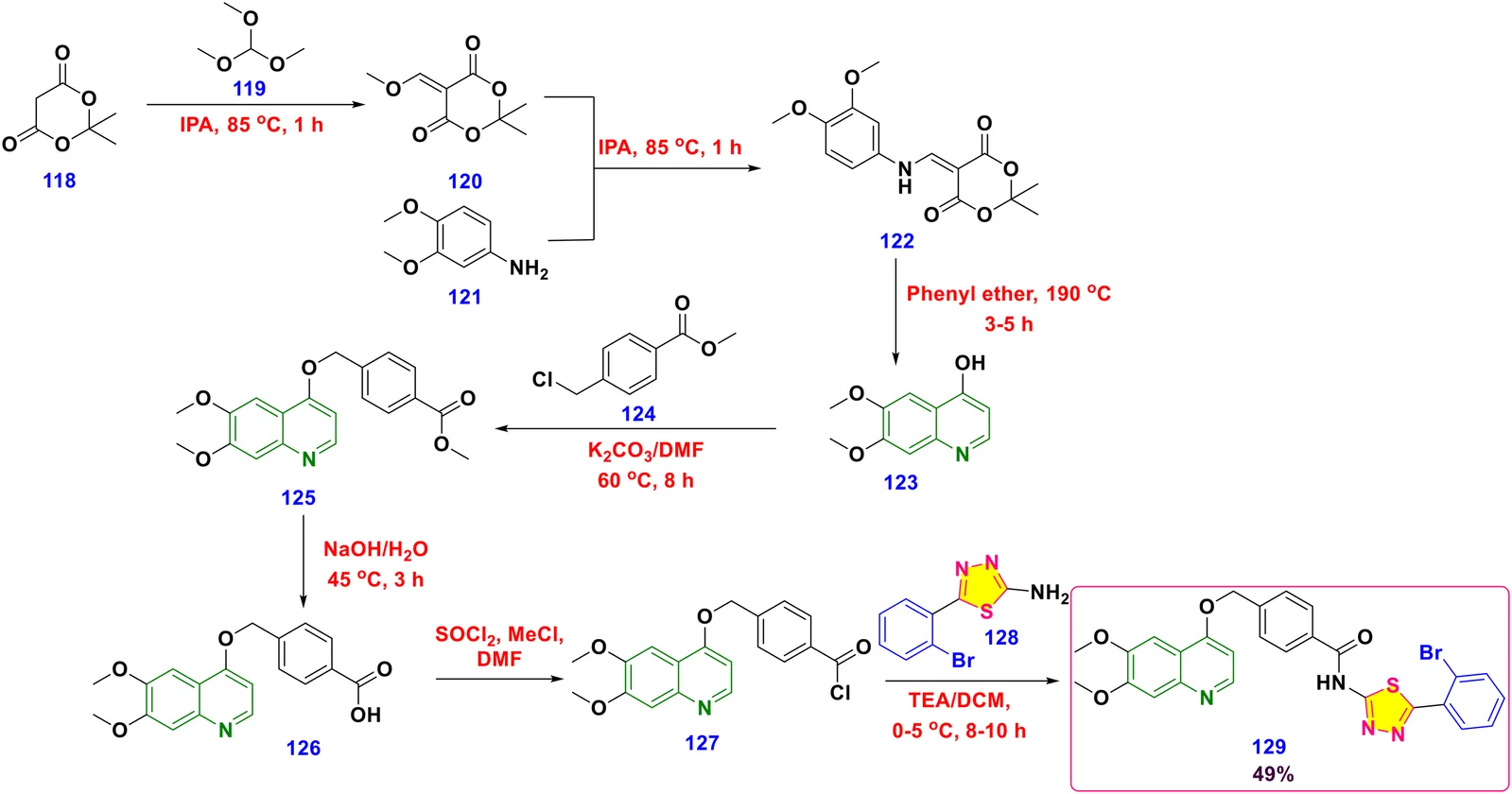
Synthesis of the quinoline-based thiadiazole motif 129 with EGFR inhibitory activity.

Kuna and coworkers synthesized an indole-tagged imidazo-thiadiazole analogue 133, which showed high potency against EGFR-targeting MCF-7 and A549 cancer cells. The reaction of indole-2-carboxylic acid 130 with thiosemicarbazide 26 in POCl_3_ furnished the indole-thiadiazole amine 131, whose subsequent treatment with 4-nitrophenacyl bromide 132 afforded the target compound 133 with a fused imidazo-thiadiazole moiety. It demonstrated good activity with IC_50_ values of 7.31 ± 0.17 and 6.89 ± 0.37 μM against MCF-7 and A549 cancer cells, respectively, compared with doxorubicin. The selectivity indices of 11.79 and 12.51 towards MCF-7 and A549 cancer cells, respectively, truly favoured the therapeutic potential of the compound. It showed an IC_50_ value of 5.66 ± 0.25 μM against EGFR kinase in comparison with erlotinib. Also, the obedience with Lipinski's rule of five gives a preliminary indication for oral bioavailability prior to further several screening tests. Molecular docking results displayed H-bonding with Asp813, Arg845 and Tyr845 residues locked in the EGFR active site. The docking poses showed these interactions when the final compound was docked with the protein (PDB ID: 4HJO) using the AutoDock tool (Scheme 21).^62^

**Scheme 21:**
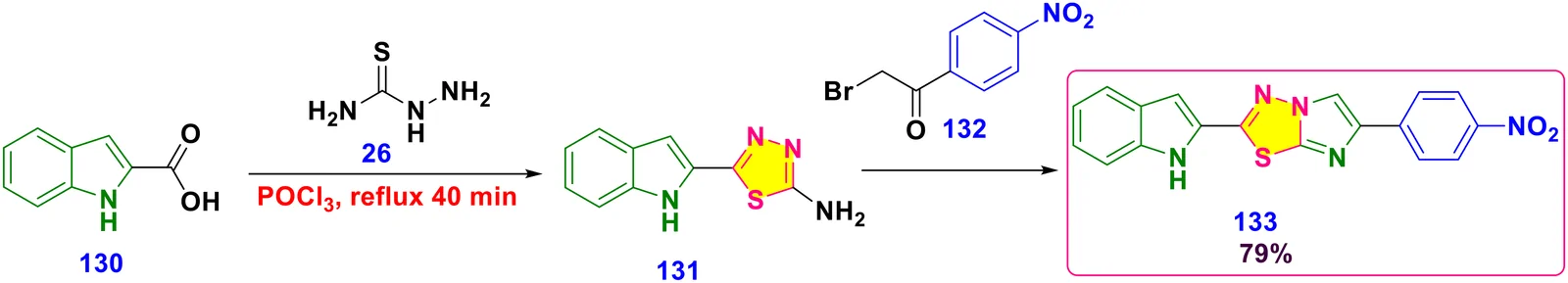
Synthesis of the indole-based imidazo-thiadiazole analogue 133.

Rasgania and colleagues developed the isatin-clubbed thiadiazole 141 by acetylating isatin 134 to get the *N*-acetyl derivative 135, which serves as the intermediate for reacting with compound 140. 140 was synthesized *via* the esterification of 2-chlorobenzoic acid 136 to the corresponding ester 137, which was converted to its hydrazide 138 using hydrazine hydrate; this molecule was then treated with KSCN and H_2_SO_4_ to form the thiadiazole-cyclized product 140. The intermediate 135 was then reacted with 140 to give the lead molecule 141. Further, upon investigating its anticancer potential, it illustrated good activity against EGFR-targeted MDA-MB-231 cancer cells with an IC_50_ value of 57.79 μM compared with the standard drug tamoxifen citrate. It obeyed Lipinski's rule of five and possessed drug-likeness, making it a potential candidate for developing anticancer drug molecules. Molecular docking showed strong H-bonds with Phe856, Asp855 and Met793 residues bound within the binding region of EGFR. These results were achieved after docking the final compound with the protein (PDB ID: 3PO2) using the AutoDock software (Scheme 22).^63^

**Scheme 22:**
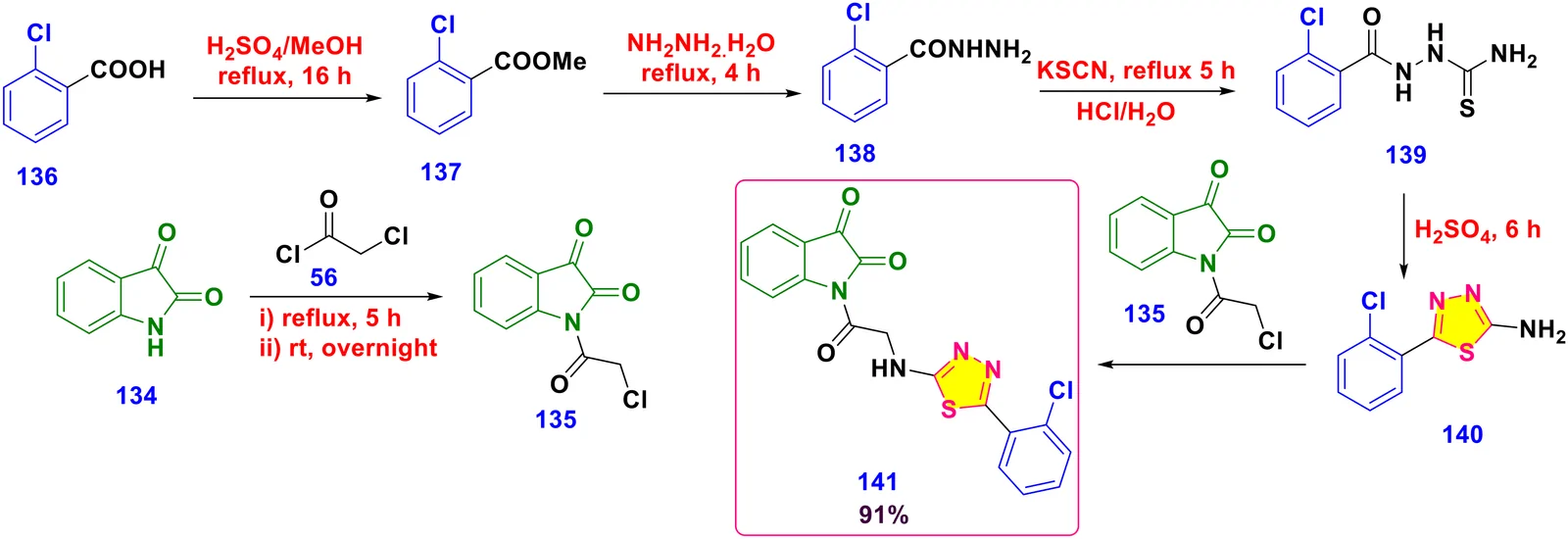
Synthesis of the isatin-based thiadiazole hybrid 141 with EGFR inhibitory activity.

Abouzied and coworkers evaluated the anticancer efficacy of the pyridine-linked thiadiazole hybrid 145. 1-Amino-4,6-dimethyl-2-oxo-1,2-dihydropyridine-3-carbonitrile 142 and phenyl isothiocyanate 66 were reacted to give an intermediate 143, which was then treated with another substrate 144 to give a thiadiazole-pyridine active agent 145. Compound 145 showed significant anti-proliferative activity against HCT-116 colon and HepG2 hepatocellular cancer cells, as evidenced by IC_50_ values of 2.03 ± 0.72 and 2.17 ± 0.83 μM, respectively, compared to the standard reference drug harmine and targeted EGFR kinase. It displayed EGFR inhibition with an IC_50_ value of 3.22 ± 0.10 μM in comparison with erlotinib. Molecular docking showed H-bond interactions with Arg841, Asn842, Lys745 and Thr854 when docked with the protein (PDB ID: 1M17) using the MOE2019 software (Scheme 23).^64^

**Scheme 23:**
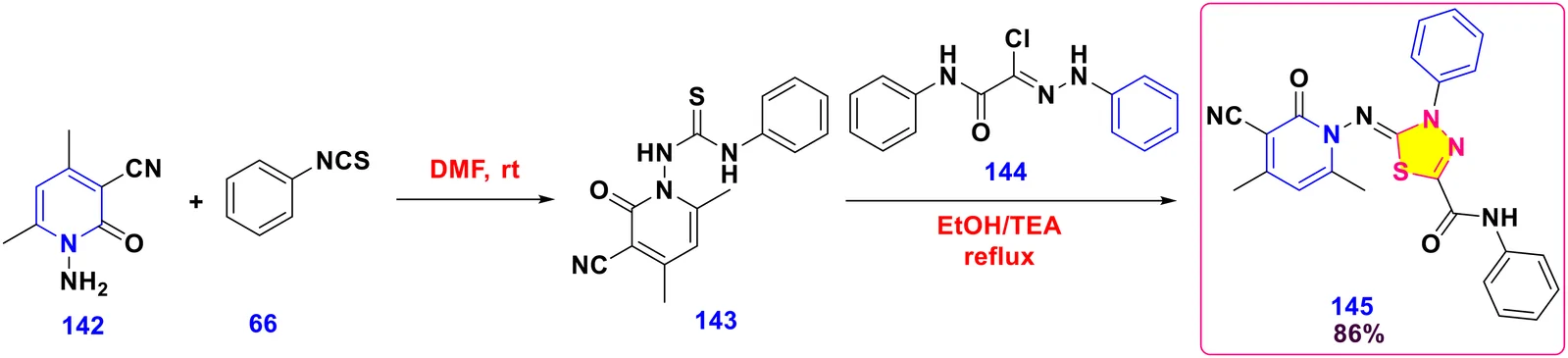
Synthesis of the pyridine-linked thiadiazole hybrid 145.

An overview of the thiadiazole-containing heterocyclic molecules with EGFR kinase inhibitory activity is provided in Table 4.

**Table 4:** Overview and comparison of the EGFR kinase inhibition activity of different thiadiazole-containing heterocyclic hybrids

Compound	Substituent	Major interactions	Observed SAR trends	EGFR: IC_50_ value/% inhibition	Reference EGFR inhibitor	Cancer cell line	Limitations	Reference
106	4-Bromophenyl substituent attached to the imidazo-thiadiazole fused ring system	Strong H-bonds with Asp831 and Thr830 within the binding pocket of EGFR	Electron-withdrawing bromo substituent and its hydrophobicity improved the cytotoxicity with the inhibition of EGFR kinase	5.04 ± 0.14 μM	Erlotinib: 4.67 ± 0.27 μM	MCF-7: 8.55 ± 0.73 μM; A549: 5.89 ± 0.32 μM	Advanced pharmacokinetic validation is required	58
110	4-Iodobutyl chain linked to the thiol group of thiadiazole	H-Bonds with Lys721, Met769 and Val702 within the EGFR active site	Isoindoline group increased the anticancer effect along with the aliphatic iodobutyl chain	6.01 ± 0.10 μM	Erlotinib: 4.22 ± 0.13 μM	PC3: 21.17 ± 7.59 μM	Lacks *in vivo* animal tumor models or pharmacokinetic/pharmacodynamic validation	59
117	Glucopyranosyl group attached to the triazole ring	H-Bonds with Met769, Met793 and Met801 within the EGFR binding pocket	Replacement with deacetylated groups significantly increased the cytotoxicity	0.5 μM	Erlotinib: 0.13 μM	HCT-116: 2.2 μM	*In vivo* animal model testing is required	60
129	2-Bromophenyl group attached to the thiadiazole ring	H-Bonds with Asp831, Val702 and Thr766 residues found to be embedded within the EGFR active binding site	Hydrophobic interactions and electron-withdrawing bromo substituents tended to increase the cytotoxicity	29.30 ± 3.28 μM	Cabozantinib: 0.96 ± 0.324 μM	A549: 1.03 ± 0.37 μM; MCF-7: 1.45 ± 0.19 μM	Lacks long-term systemic toxicity and biodistribution profiling studies	61
133	4-Nitrophenyl substituent attached to the imidazo-thiadiazole scaffold	H-Bonding with Asp813, Arg845 and Tyr845 residues locked in the EGFR active site	An electron-withdrawing nitro group tended to increase the cytotoxicity linked with the phenyl moiety	5.66 ± 0.25 μM	Erlotinib: 3.48 ± 0.14 μM	MCF-7: 7.31 ± 0.17 μM; A549: 6.89 ± 0.37 μM	Advanced pharmacokinetic evaluations are missing in this study	62
141	2-Chlorophenyl substituent linked to the thiadiazole molecule	Strong H-bonding with Phe856, Asp855 and Met793 residues bound within the EGFR binding region	Hydrophobic 2-chlorophenyl substituent improved the cytotoxicity	57.79 μM	Erlotinib: 7.26 μM	MDA-MB-231: 72.35 μM	Low EGFR inhibition activity compared with erlotinib	63
145	Pyridine and phenyl groups linked to the thiadiazole moiety	H-bond interactions with Arg841, Asn842, Lys745 and Thr854	Electron-withdrawing amido group along with a phenyl ring increased the anticancer efficacy	3.22 ± 0.10 μM	Erlotinib: 2.40 ± 0.12 μM	HCT-116: 2.03 ± 0.72 μM; HepG2: 2.17 ± 0.83 μM	Further studies on *in vivo* models are to be carried out	64

### 2.3 SHP2 inhibitors

Liu *et al.* produced a thiadiazole-containing spirocyclic compound 150. 2,5-dibromo-1,3,4-thiadiazole 146 and (3*S*,4*S*)-3-methyl-2-oxa-8-azaspiro[4.5]decan-4-amine 147 were reacted to generate an *N*-thiadiazole scaffold 148, and its subsequent treatment with 2,3-dichlorothiophenol 149 using CuI as the catalyst in the presence of 1,10-phenanthroline yielded the final compound 150. This compound showed good activity in inhibiting SHP2 with an IC_50_ value of 0.25 ± 0.02 μM. Additionally, it showed promising inhibition against SHP2-E76K and SHP2-E76A with IC_50_ values of 6.88 ± 0.69 μM and 1.38 ± 0.12 μM, respectively. When this compound was tested against MV4-11 and NCI-H358 cells, it showed IC_50_ values of 3.85 ± 0.34 μM and 12.01 ± 0.62 μM, respectively, when compared with the SHP2 allosteric inhibitor SHP099. Furthermore, the compound was found to obey Lipinski's rule of five and other screening parameters. Molecular docking studies revealed several interactions, such as strong H-bonds with Thr108, Arg111 and Phe113 in the allosteric region of SHP2. These docking interactions were obtained when the final compound was subjected to computational docking with the protein (PDB ID: 5EHR) using the GROMACS tool (Scheme 24).^65^

**Scheme 24:**
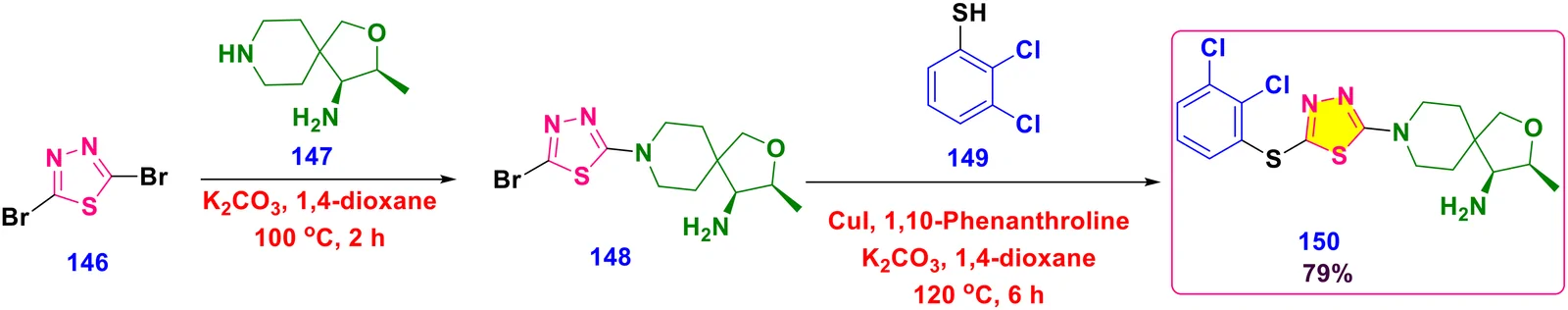
Synthesis of the thiadiazole-containing spirocyclic compound 150.

Mitra and coworkers constructed a thiadiazole-tethered triazole hybrid 157 with remarkable efficacy in inhibiting SHP2 and potential for emerging as a new drug candidate. The synthesis started by producing triazole and thiadiazole and combining them in the final step. First, 4-methoxybenzoic acid 151 was treated with thiosemicarbazide in the presence of oxalyl chloride to give the thiosemicarbazate salt 152, and then, it was reacted with KOH and HCl by maintaining a pH of 5–6 to give the intermediate 153. Second, benzo[*d*][1,3]dioxole-5-carboxylic acid 154 was treated with thiosemicarbazide, refluxed with water and neutralized using 40% NaOH to produce 155, which upon acetylating with chloroacetyl chloride and base treatment produced another intermediate 156. Finally, combining these two intermediates led to the formation of the target molecule 157. This molecule showed good cytotoxicity and inhibitory action against SHP2 with an IC_50_ value of 0.318 ± 0.001 μM, and this lead molecule was bound to the tunnel allosteric site of SHP2. Furthermore, it inhibited tumour growth by 50% with a GI_50_ value of 37.02 ± 0.25 μM against MCF-7 cancer cells with reference to SHP099. Molecular docking studies showed that both the thiadiazole and triazole rings form bonds with Arg111. Moreover, the thiadiazole ring makes a bond with Lys492 and the triazole ring makes an H-bond with Leu216, and all these interactions were furnished after docking the final compound with the protein (PDB ID: 5EHR) using the AutoDock tool (Scheme 25).^66^

**Scheme 25:**
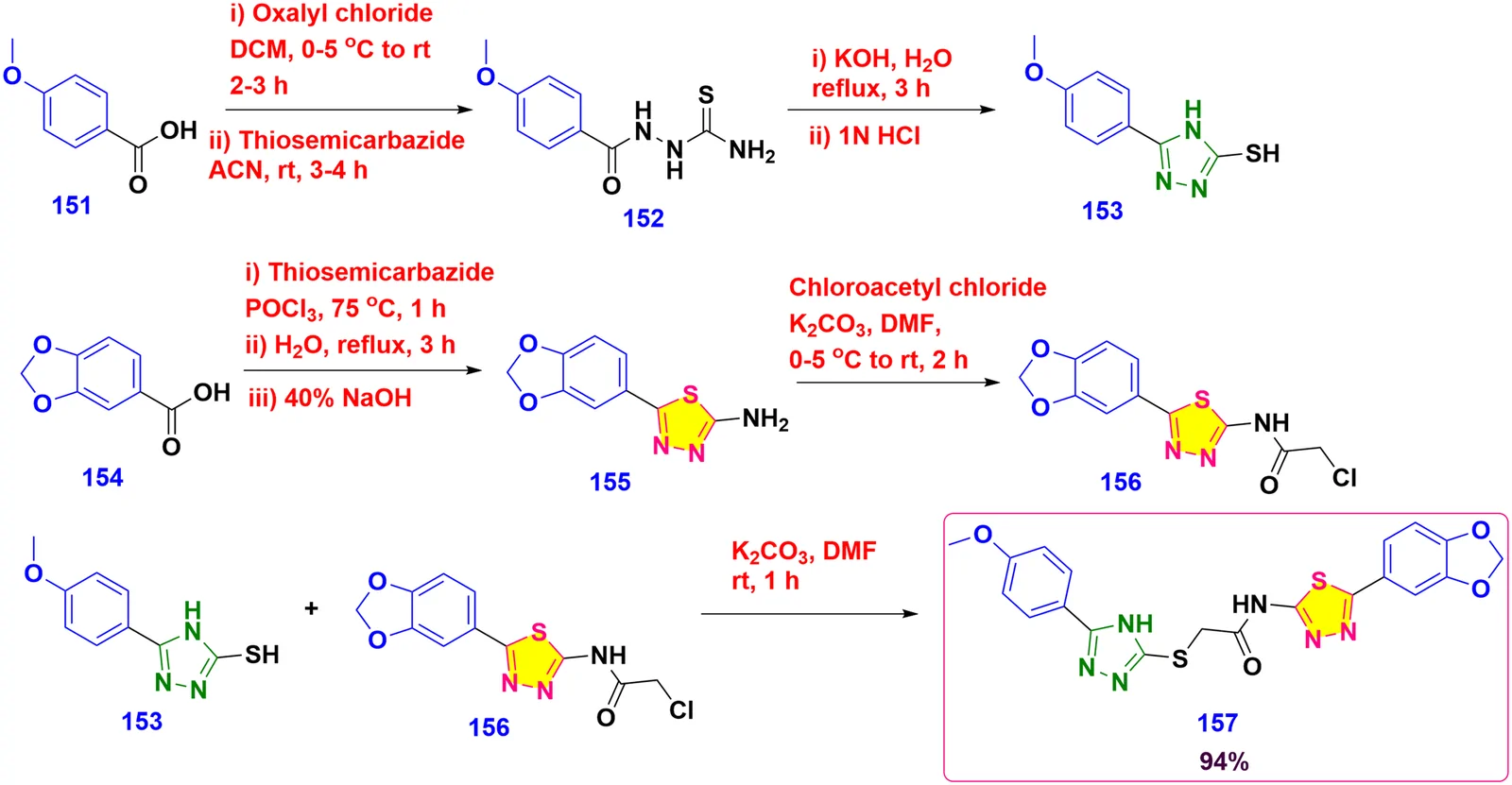
Synthesis of the SHP2 inhibitor thiadiazole-tethered triazole 157.

Ayyannan *et al.* designed acetophenylhydrazones of thiadiazoles 163. 5-Methyl-1,3,4-thiadiazole-2-thiol 158 and ethyl-2-bromoacetate 159 were reacted under freezing conditions and then at room temperature for 1–2 h to yield the corresponding thioester 160, and its conversion into the thiohydrazide 161 was performed using hydrazine, followed by treatment with 2,6-dichlorobenzaldehyde 162 to form the Schiff's base lead compound 163. This lead molecule 163 was a potent SHP2 inhibitor with an IC_50_ value of 0.120 ± 0.006 μM compared to SHP099 and exhibited remarkable cytotoxicity against MCF-7 human breast cancer cells with an IC_50_ value of 86.46 ± 2.32 μM compared to docetaxel in a dose-dependent manner. Additionally, it exhibited a good safety profile in mice at an oral dose administration of 2000 mg kg^−1^ BW. Docking studies revealed strong interactions of the compound with Glu250, Arg229, Thr218 and Glu110. His114 showed H-bonding with the allosteric triad of SHP2 after docking the final compound with the protein (PDB ID: HER) using the AutoDock software (Scheme 26).^67^

**Scheme 26:**
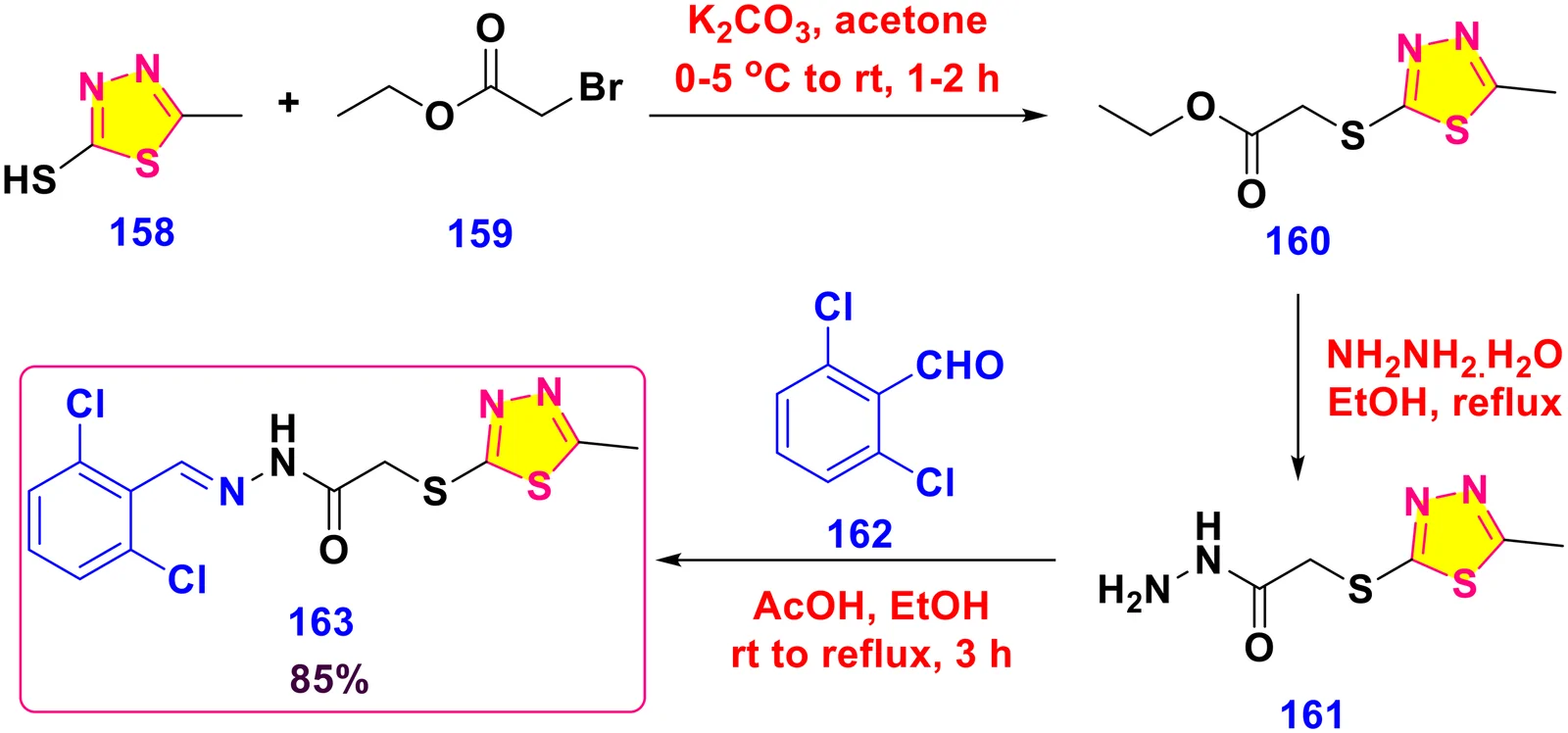
Synthesis of the acetophenylhydrazones of thiadiazole 163 with SHP2 inhibitory activity.

Zhang *et al.* synthesized an imidazo-thiadiazole acid derivative 168. (*E*)-3-(*p*-Tolyl)acrylic acid 164 and thiosemicarbazide 26 were reacted to give the vinylic thiadiazole 165, which was treated with methyl 5-(2-bromoacetyl)-2-hydroxybenzoate 166 to generate the imidazo-thiadiazole ester 167, whose hydrolysis using LiOH furnished the corresponding acid derivative 168. Its anticancer activity with the inhibition of SHP2 was revealed by its IC_50_ value of 2.89 ± 1.60 μM and evidenced that it was effective in blocking the SHP2-mediated p-ERK signalling pathway. Additionally, it showed prominent green fluorescence imaging in HeLa cells and zebrafish. The scope for designing such a fluorescent SHP2 inhibitor showcased the potential of organic heterocyclic thiadiazole molecules in future applications. It also inhibited the proliferation in MV4-11 cancer cells with an IC_50_ value of 7.90 ± 0.75 μM. Molecular docking showed that π-alkyl interactions with P312 enhanced the ligand–protein interaction. H-Bond interaction with Q347 and π–S interaction with M311 are termed as the key interactions for SHP2 inhibitory activity after docking the target compound with the protein (PDB ID: 3O5X) using the AutoDock tool (Scheme 27).^68^

**Scheme 27:**
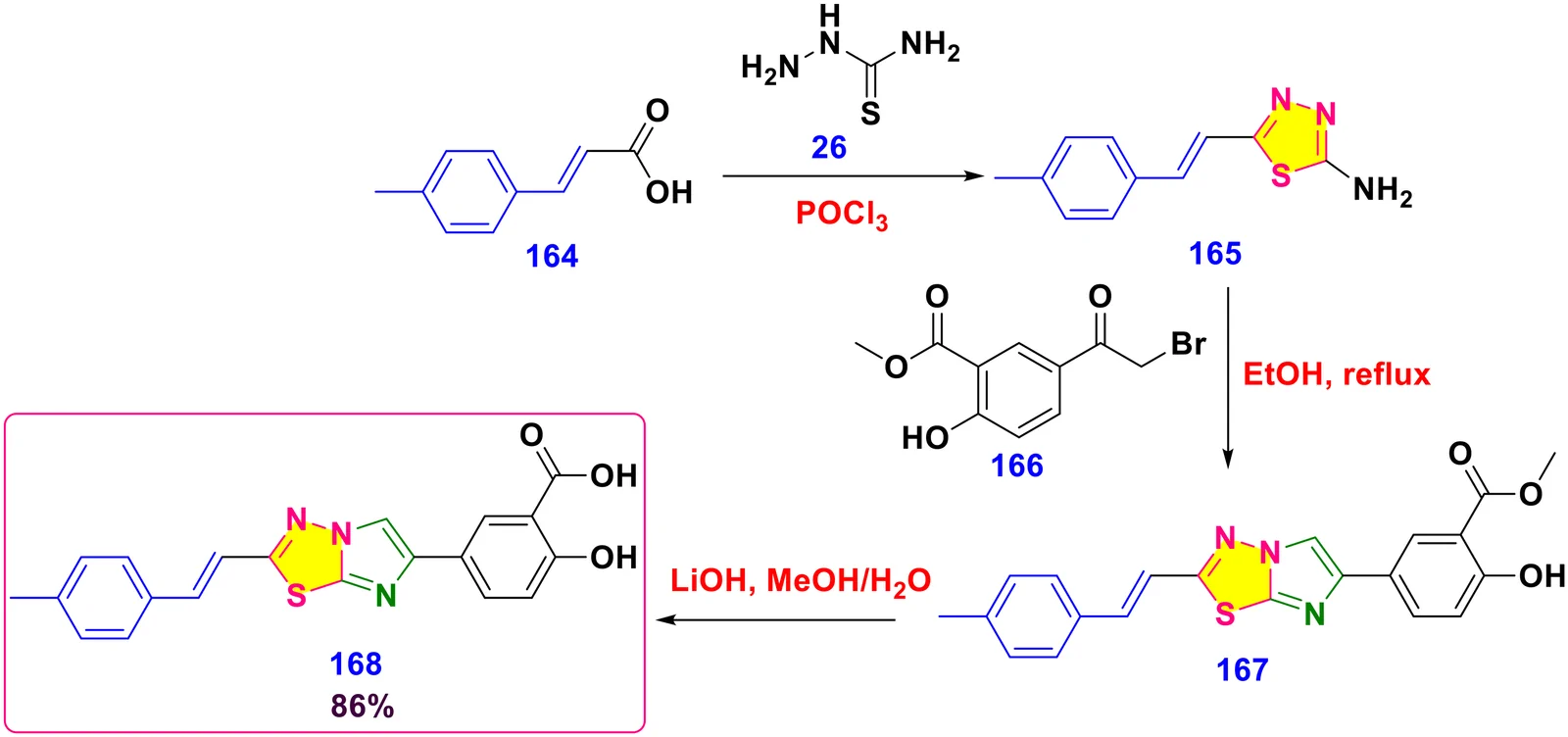
Synthesis of the imidazo-thiadiazole acid derivative 168.

An overview of the thiadiazole-containing heterocyclic molecules with SHP2 inhibitory activity is provided in Table 5.

**Table 5:** Overview and comparison of the SHP2 inhibitory activity of different thiadiazole-containing heterocyclic hybrids

Compound	Substituent	Major interactions	Observed SAR trends	SHP2: IC_50_ value/% inhibition	Reference SHP2 inhibitor	Cancer cell line	Limitations	Reference
150	2,3-Dichlorothiophenyl substituent linked to the thiadiazole moiety	Strong H-bond interactions with Thr108, Arg111 and Phe113 in the allosteric region of SHP2	2,3-Dichloro groups significantly increased inhibitory action against SHP2	0.25 ± 0.02 μM	SHP2 allosteric inhibitor SHP099: 0.085 ± 0.007 μM	MV4-11: 3.85 ± 0.34 μM; NCI-H358: 12.01 ± 0.62 μM	*In vivo* animal model evaluations are lacking in this study	65
157	4-Anisyl ring attached to triazole; benzo[*d*][1,3]dioxole group attached to the thiadiazole ring	Both the thiadiazole and triazole rings make bonds with Arg111; the thiadiazole ring makes a bond with Lys492; the triazole ring makes an H-bond with Leu216	The electron-donating 4-anisyl group tended to increase the cytotoxicity offered by the thiadiazole	0.318 ± 0.001 μM	SHP2 allosteric inhibitor SHP099: 0.070 μM	MCF-7: 37.02 ± 0.25 μM (GI_50_)	Pharmacokinetic aspects are not covered	66
163	2,6-Dichlorophenyl hydrazone moiety linked to the thiadiazole ring	Strong interactions of the compound with Glu250, Arg229, Thr218 and Glu110	Two electron-withdrawing groups significantly increased the cytotoxicity index	0.120 ± 0.006 μM	SHP2 allosteric inhibitor SHP099: 0.070 μM	MCF-7: 86.46 ± 2.32 μM	Moderate cellular potency is observed, and *in vivo* studies are required for further validation of the lead compound	67
		His114 showed H-bonding with the allosteric triad of SHP2						
168	4-Tolyl group attached through a vinylic bond to the thiadiazole ring	π-Alkyl interactions with P312 enhanced the ligand-protein interaction. H-Bond interaction with Q347 and π–S interaction with M311 were termed as the key interactions for SHP2 inhibitory activity	Electron-donating tolyl group improved the activity	2.89 ± 1.60 μM	SHP2 allosteric inhibitor SHP099: 0.071 μM	MV4-11: 7.90 ± 0.75 μM	Higher water concentrations led to fluorescence quenching; poor aqueous solubility; further *in vivo* animal model testing is required	68

## 3. Conclusion

1,3,4-Thiadiazole compounds have emerged as interesting candidates due to their anticancer efficacy. Various 1,3,4-thiadiazole-based heterocyclic motifs have significantly inhibited major tyrosine kinase enzymes like VEGFR-2 and EGFR. Their potential in this remarkable inhibitory action tends to broaden the scope for developing newer molecules with susceptible and active linkers targeting VEGFR-2 and EGFR kinases. Most of the discussed compounds were found to be more active than the standard reference drugs used to treat different types of cancer based on the preliminary anticancer evaluation studies. Furthermore, their drug-likeness, compliance with Lipinski's rule of five, higher selective indices and other cytotoxicity factors pave the way for developing and constructing thiadiazole hybrids with anticancer activity. Several thiadiazole-containing compounds have demonstrated target inhibition in the low micromolar to nanomolar range, meaningful antiproliferative activity, and in selected cases, favourable selectivity towards cancer cells over normal cells. The biological performance of these scaffolds can be substantially modulated through the strategic incorporation of hydrophobic aryl groups, electron-donating or electron-withdrawing substituents, heteroaromatic rings, flexible linkers, and pharmacophoric motifs capable of establishing H-bonding and hydrophobic interactions within the kinase or phosphate binding sites. The emergence of thiadiazole-containing compounds as SHP2 inhibitors is particularly noteworthy as it expands their utility beyond the conventional kinase-directed cancer therapies and provides an opportunity to interfere with oncogenic signaling through complementary molecular modes. The synthesis of thiadiazole compounds has been instrumental in both industry and academia. Compared with conventional methods, the use of green methods like microwave-assisted and ultrasound techniques would help in carrying out chemical reactions in a sustainable and eco-friendly way.

Most of the thiadiazole compounds discussed in this review have been shown to be potent in their action, and additional insights from their molecular docking studies, such as major interactions and SAR observations, will be crucial in the future for their production. Electron-donating and electron-withdrawing groups play a vital role in increasing the cytotoxicity and anticancer efficacy. The hydrophobic nature of the scaffolds has been an interesting factor of study recently to generate compounds with more activity and anticancer performance. Nevertheless, the current field remains predominantly at the hit-to-lead and preclinical discovery stages. A major limitation is that high potency in isolated enzyme assays or cancer-cell models does not necessarily translate into therapeutic efficacy. Many of the reported compounds are supported primarily by *in vitro* cytotoxicity, target enzyme inhibition, molecular docking, and preliminary drug-likeness assessments. Although Lipinski compliance and favourable selectivity indices provide useful early indicators, they cannot substitute the systematic evaluations of pharmacokinetics and metabolic stability.

In the coming years, the scope of developing new thiadiazole-based heterocyclic systems and transforming them into drug candidates is wide and bright. The strong fundamentals of medicinal chemistry enable chemists working in the research and industrial sectors to generate biologically active pharmacophores like thiadiazole molecules. For EGFR and VEGFR-2 inhibitory activity, future research should prioritize isoform- and mutant-selective inhibitors *via* the rational exploitation of the non-conserved regions adjacent to the ATP-binding pocket. Type-II and allosteric binding strategies may offer additional opportunities to improve selectivity and overcome the limitations associated with the highly conserved ATP sites. For SHP2 inhibition, the demonstrated activity of thiadiazole-containing spirocyclic, triazole-linked, hydrazone, and imidazo-thiadiazole architectures suggests that further exploration of allosteric and tunnel-binding pockets could yield chemically differentiated inhibitors. Finally, future thiadiazole-based drug discovery research should incorporate ADME-based design, metabolic liability assessment, green and scalable synthesis, advanced computational modelling, and rigorous *in vivo* validation from the earliest stages of lead optimization.

## Author contributions

Glanish Jude Martis: writing – original draft, software. Santosh L. Gaonkar: writing – review and editing, supervision.

## Conflicts of interest

The authors declare no competing financial interests.

## Data Availability

All data were obtained from the peer-reviewed articles cited in the reference list, with no additional datasets utilized.
